# An automated framework for NMR chemical shift calculations of small organic molecules

**DOI:** 10.1186/s13321-018-0305-8

**Published:** 2018-10-26

**Authors:** Yasemin Yesiltepe, Jamie R. Nuñez, Sean M. Colby, Dennis G. Thomas, Mark I. Borkum, Patrick N. Reardon, Nancy M. Washton, Thomas O. Metz, Justin G. Teeguarden, Niranjan Govind, Ryan S. Renslow

**Affiliations:** 1 The Gene and Linda Voiland School of Chemical Engineering and Bioengineering, Washington State University, Pullman, WA USA; 2 Earth and Biological Sciences Division, Pacific Northwest National Laboratory, Richland, WA USA; 3 Nuclear Magnetic Resonance Facility, Oregon State University, Corvallis, OR 97331 USA

**Keywords:** Chemical shift, Density functional theory, DFT, Metabolomics, NMR, NWchem, Python, Quantum chemistry

## Abstract

When using nuclear magnetic resonance (NMR) to assist in chemical identification in complex samples, researchers commonly rely on databases for chemical shift spectra. However, authentic standards are typically depended upon to build libraries experimentally. Considering complex biological samples, such as blood and soil, the entirety of NMR spectra required for all possible compounds would be infeasible to ascertain due to limitations of available standards and experimental processing time. As an alternative, we introduce the in silico Chemical Library Engine (ISiCLE) NMR chemical shift module to accurately and automatically calculate NMR chemical shifts of small organic molecules through use of quantum chemical calculations. ISiCLE performs density functional theory (DFT)-based calculations for predicting chemical properties—specifically NMR chemical shifts in this manuscript—via the open source, high-performance computational chemistry software, NWChem. ISiCLE calculates the NMR chemical shifts of sets of molecules using any available combination of DFT method, solvent, and NMR-active nuclei, using both user-selected reference compounds and/or linear regression methods. Calculated NMR chemical shifts are provided to the user for each molecule, along with comparisons with respect to a number of metrics commonly used in the literature. Here, we demonstrate ISiCLE using a set of 312 molecules, ranging in size up to 90 carbon atoms. For each, calculation of NMR chemical shifts have been performed with 8 different levels of DFT theory, and with solvation effects using the implicit solvent Conductor-like Screening Model. The DFT method dependence of the calculated chemical shifts have been systematically investigated through benchmarking and subsequently compared to experimental data available in the literature. Furthermore, ISiCLE has been applied to a set of 80 methylcyclohexane conformers, combined via Boltzmann weighting and compared to experimental values. We demonstrate that our protocol shows promise in the automation of chemical shift calculations and, ultimately, the expansion of chemical shift libraries.

**Electronic supplementary material:**

The online version of this article (10.1186/s13321-018-0305-8) contains supplementary material, which is available to authorized users.

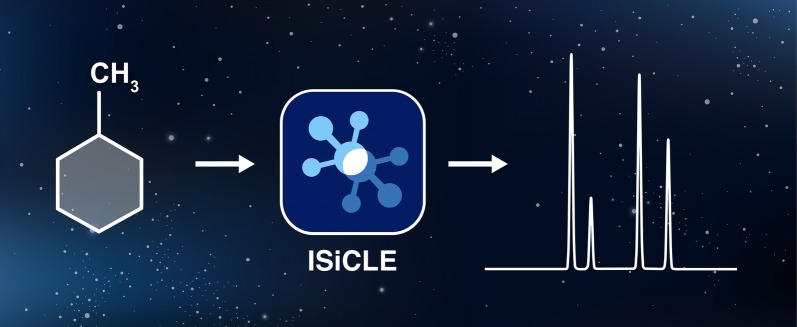

## Background

Metabolomics is being increasingly applied in biomedical and environmental studies, despite the technical challenges facing comprehensive and unambiguous identification of detected metabolites [1–3]. The capability to routinely measure and identify even a modicum of biologically important molecules within all of chemical space—greater than 10^60^ compounds [4]—remains a grand challenge in biology. The prevention and treatment of metabolic diseases, determining the interactions between plant and soil microbial communities, and uncovering the building blocks that led to abiogenesis will all strongly depend on confidently identifying small molecules, and thus understanding the mechanisms involved in the complex processes of metabolic networks [5–7]. The current gold standard for chemical identification requires matching chemical features to those measured from an authentic chemical standard. However, this is not the case with the vast majority of molecules. For example, only 17% of compounds found in the Human Metabolome Database (HMDB) and less than 1% of compounds found in exposure chemical databases like the U.S. Environmental Protection Agency (EPA) Distributed Structure-Searchable Toxicity (DSSTox) Database [8] can be purchased in pure form [9, 10]. Although analytical techniques like nuclear magnetic resonance (NMR) spectroscopy [11–13] and mass spectrometry (MS) [14–16] have been applied for the identification of metabolites and to build libraries [17–21], determining the complete composition of entire metabolomes is still non-trivial for both technical and economic reasons. In this regard, libraries constructed of experimentally obtained data are too limited, expensive, and slow to build, even for libraries with thousands of metabolites [22–25].

The most practical approach expand reference libraries for comprehensive identification of compounds detected in metabolomics studies is through in silico calculation of molecular attributes. Molecular properties that can be both accurately predicted computationally and consistently measured experimentally may be used in “standards free” metabolomics identification approaches. The metabolomics community has made many advances in calculations of measurable chemical attributes, such as chromatographic retention time [26, 27], tandem mass spectra [28–30], ion mobility collision cross section [31, 32], and NMR chemical shifts [33]. Recently, high throughput computation of chemical properties has been demonstrated using machine learning approaches [34–37]. These tools are a good resource for the metabolomics community, however, machine learning methods are limited by the size and scope of the initial training set, and thus ultimately limited by the number of authentic chemical standards available for purchase. In contrast, structure-based approaches, utilizing first principles of quantum chemical calculations, leverage our understanding of the underlying chemistry and physics to directly predict chemical properties of any chemically valid molecule. Thus, quantum chemical calculations enable us to overcome the reliance on authentic chemical standards in metabolomics. In this study, we focus on expanding the utility of density functional theory (DFT), a widely used electronic structure approach, which has been applied to predict NMR chemical shifts [38–41]. DFT enables examination of molecular conformers [42–45] and allows custom solvent conditions [46–48]. Ultimately, computational modeling can be used in the rapid identification and study of thousands of metabolites, culminating in in silico metabolome libraries of multiple chemical properties. Furthermore, the same tools that can be used to aid identification of small molecules in complex samples can also be used for structure confirmation and correction. For example, we recently used the tool described in this manuscript to help correct the misidentification of the isoflavonoid wrightiadione to the actual structure as an isobaric isostere, the alkaloid tryptanthrin [49].

Metabolomics researchers unfamiliar with DFT or similar calculations may find the application of quantum chemical calculations complicated or challenging to apply quickly, and thus avoid these techniques. To this end, and to help bring DFT calculations to large sets of small organic molecules relevant to the mainstream metabolomics community, we have developed a Python-based workflow and analysis package, the ISiCLE (in silico Chemical Library Engine) NMR chemical shift module employs DFT methods through use of NWChem [50], a high-performance quantum chemistry software package developed at Pacific Northwest National Laboratory (PNNL). The module automates calculations of NMR chemical shifts, including solvent effects, via the COnductor-like Screening Model (COSMO) [51] of user-specified NMR-active nuclei for a given set of molecules for multiple DFT methods. ISiCLE also calculates the corresponding errors if experimental values are available. In this paper, we describe ISiCLE’s NMR module, provide a working tutorial example, demonstrate its use through the calculation of chemical shifts for a large set of small molecules, and, finally, show how ISiCLE can be applied to rapidly calculate chemical shifts of arrays of Boltzmann-weighted conformers to yield high accuracy chemical shift calculations.

## Methods

### In silico Chemical Library Engine (ISiCLE)—NMR module

**Fig. 1:**
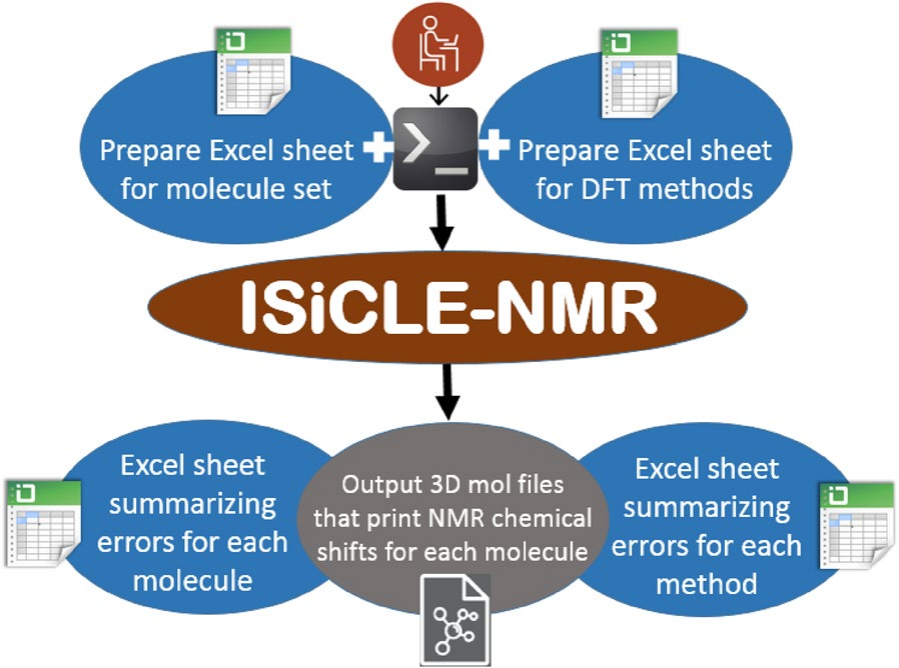
Schematic representation of inputs and outputs of the ISiCLE NMR module

ISiCLE is a Python module that provides straightforward automation of DFT using NWChem, an open source, high-performance computational quantum chemistry package, developed at Pacific Northwest National Laboratory (PNNL), for geometry optimization and chemical shift and solvent effect calculations. Figure 1 shows a schematic representation of ISiCLE. For typical use, ISiCLE requires only a list of molecules and a list of desired levels of DFT theory from the user. For more advanced use cases, users may adjust NWChem parameters by modifying the provided .nw template file.

**Fig. 2:**
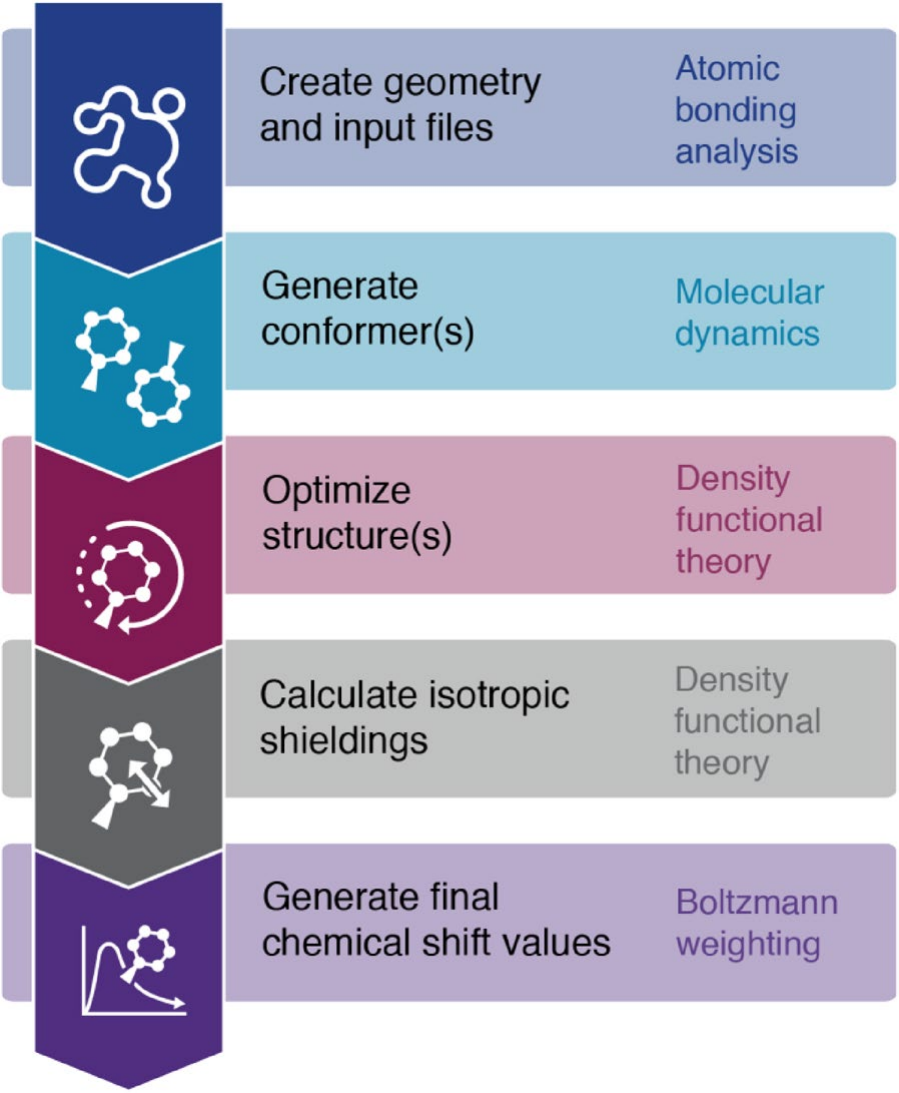
The step-by-step conceptual workflow for the ISiCLE NMR module. Conformer generation with Boltzmann weighting is optional and will be automated in subsequent versions. Please see github.com/pnnl/isicle for the latest versions

Here, we describe each step of a typical ISiCLE run (see Fig. 2 for a general workflow for using the ISiCLE NMR module).

To start, users must prepare File A, containing a list of molecules, and File B, containing a list of DFT combinations, which both are required to be in Excel format (.xls or .xlsx). File A must contain all input molecules either as (i) International Union of Pure and Applied Chemistry (IUPAC) International Chemical Identifier (InChI) strings [52, 53] or (ii) XYZ files, a free-format text file having XYZ coordinates of atoms. In subsequent versions, alternative file formats will be supported, such as TSV for inputs and outputs.

Once prepared, the user runs ISiCLE. First, ISiCLE opens File A for the input molecules. OpenBabel, an open-source chemical informatics toolbox available with Python wrappers [54, 55], is called to generate geometry files. For InChI inputs, OpenBabel generates .xyz files for each molecule, unless .xyz files are provided, and converts InChI to InChIKey for naming files (otherwise, the base names of XYZ files are used for naming subsequent files). Next, OpenBabel applies the Merck molecular force field (MMFF94) [56] to generate a rough three-dimensional (3D) structure for each molecule, resulting in associated .mol files. ISiCLE then prepares NWChem input files based on the specified DFT methods, solvents, shielding parameters and regarding task directives given by the user-prepared File B. Finally, ISiCLE submits the appropriate files to, if relevant, a remote NWChem installation (typically on a non-local, networked, high-performance computer), and then retrieves the output files once the calculations are complete. Additional information and further details about ISiCLE is provided in Additional file 1 (S1). Note that future versions of ISiCLE will automatically generate conformers of a given molecule, as part of the seamless pipeline.

For each molecule, ISiCLE generates MDL Molfiles (.mol) [57] that contain isotropic shieldings and NMR chemical shifts. ISiCLE exports isotropic shieldings for each molecule and appends them to a MDL Molfile in the same atomic order of the original XYZ files. Then, ISiCLE converts isotropic shieldings to NMR chemical shifts by subtracting the isotropic shielding constants for the specified nuclei of the molecule of interest from those of a reference compound computed at the same level of theory (Eq. 1). For this manuscript, tetramethylsilane (TMS) is used as a reference compound. The experimental chemical shifts of TMS are assigned a value of zero, thus the calculation of NMR chemical shifts needs only isotropic shieldings of TMS [58–61]. Any molecule can be used as reference in ISiCLE as long as it has the specified nuclei and its experimental (or calculated) chemical shifts are supplied. It is explained in detail in a supplemental tutorial how a user inputs experimental data. The equation for calculating chemical shifts from isotropic shieldings is:

1 $$ \delta_{i} = \sigma_{ref} - \sigma_{i} + \delta_{ref} $$

where $\delta_{i}$ and $\delta_{ref}$ are the chemical shifts of atom *i* (of the molecule of interest) and the reference molecule, respectively. $\sigma_{i}$ and $\sigma_{ref}$ are the isotropic shielding constants of atom *i* and the reference molecule, respectively.

ISiCLE also calculates errors in NMR chemical shifts if experimental data is provided in the MDL Molfiles in a required way as explained in the tutorial. The errors are quantified in terms of mean absolute error (MAE) (Eq. 2), corrected mean absolute error (CMAE) (Eq. 3), root mean square error (RMSE) (Eq. 4), and maximum absolute error (Eq. 5).

2 $$ MAE = \frac{{\mathop \sum \nolimits_{i = 1}^{N} \left| {\delta_{exp} - \delta_{calc} } \right|}}{N} $$

3 $$ CMAE = \frac{{\mathop \sum \nolimits_{i = 1}^{N} \left| {\delta_{exp} - (\delta_{calc} - intercept)/slope} \right|}}{N} $$

4 $$ RMSE = \sqrt {\frac{{\mathop \sum \nolimits_{i = 1}^{N} \left( {\delta_{exp} - \delta_{calc} } \right)^{2} }}{N}} $$

5 $$ \mathop {\hbox{max} }\limits_{i = 1,2, \ldots ,N} \left| {\delta_{exp} - \delta_{calc} } \right| $$

where N is the total number of chemical shifts, and $\delta_{calc}$ and $\delta_{exp}$ are the lists of calculated and experimental chemical shits, respectively.

Empirical scaling of isotropic shieldings or NMR chemical shifts is the most common approach to remove systematic errors. If experimental data is provided, ISiCLE uses two optional approaches for its linear regression method, where slope and intercept values are derived from (i) regression of computed NMR chemical shifts versus experimental NMR chemical shifts using (Eq. 6), and/or (ii) regression of computed isotropic shieldings versus experimental NMR chemical shifts using (Eq. 7).

6 $$ \delta_{exp} = \frac{{intercept - \delta_{calc} }}{ - slope} $$

7 $$ \delta_{exp} = \frac{{intercept - \sigma_{calc} }}{ - slope} $$

where $\sigma_{calc}$ is the list of isotopic shielding constants of molecules.

Alternatively, if the user does not provide experimental NMR chemical shifts, ISiCLE can scale NMR chemical shifts using provided intercept and slope values. The scaled NMR chemical shifts are appended to MDL Molfiles.

A detailed description of InChIs and InChIKeys, and why they were chosen, can be found in the Additional file 1 (S2). Similarly, justification for the use of MDL Molfiles is explained in Additional file 1 (S2). In the next version, ISiCLE will be compatible with other file formats, such as the NMReDATA [62] format that has been recently designed for NMR data use. To help ease the use of our data, we provide NMReDATA files for the demonstration set in the Additional file 2.

Furthermore, installation details for OpenBabel and other required Python packages are provided in the tutorial (see Additional file 2). The Windows-based tutorial provides step-by-step instructions for running ISiCLE for the first time, including information for installation of packages, properly preparing input files, running a calculation, and obtaining output files. The tutorial includes example molecules with anticipated output files for use as a practice set and for benchmarking purposes. It is designed to guide users of ISiCLE and NWChem in the use of the input files and scripts, demonstrated using three small molecules: methanol, methyl-isothiocyanate, and nitromethane. Calculation time may vary (depending on network speed, local computational power, etc.), but it is expected to take less than 10 min.

### Demonstration set

For an initial demonstration of ISiCLE, we have compiled a molecule set of 312 compounds from previous studies: Alver [63], Asiri et al. [64], Bally and Rablen [65], Bagno et al. [66], Borkowski et al. [67], Coruh et al. [68], Fulmer et al. [69], Hill et al. [70], Izgi et al. [71], Karabacak et al. [72], Krishnakumar et al. [73–75], Kwan and Liu [45], Li et al. [76], Lomas [77], Osmialowski et al. [78], Parlak et al. [79], Perez et al. [80], Rablen et al. [81], Sarotti and Pellegrinet [82, 83], Sebastian et al. [84], Seca et al. [52], Senyel et al. [85, 86], Sridevi et al. [87], Tormena and da Silva [88], Vijaya and Sankaran [89], Watts et al. [53], Wiitala et al. [90, 91], Willoughby et al. [92], and Yang et al. [93]. We aimed to cover a broad chemical space and distribution of sizes. Our criteria also included the existence of all ^1^H and/or ^13^C NMR experimental data in chloroform solvent, referenced to TMS at room temperature, for comparisons. Note that the NMR spectra of each molecule set were not recorded at the same magnetic field strengths. A summary of the demonstration set compounds are given in Table 1. Detailed information about the individual sets is given in Additional file 1 (S3).

**Table 1:** Demonstration set sources and details

References	# of molecules	Ave. atoms per molecule	Ave. H per molecule	Ave. C per molecule	Types of molecules
Alver [63]	1	24	11	8	Boron-based compound
Asiri et al. [64]	1	33	12	16	Organic photochromic compound
Bagno et al. [66]	4	44	21	18	Small organic molecules with constrained conformations
Barkowski et al. [67]	15	83	50	31	Pentacyclic terpenoids (fernenes)
Coruh et al. [68]	1	25	8	13	Heterocyclic aromatic compound
Fulmer et al. [69]	33	14	9	4	Commonly used NMR solvents
Hill et al. [70]	1	70	33	30	Complex drug with multiple chemical groups and one stereocenter
Izgi et al. [71]	1	24	15	8	Molecule with cyclohexene (C_6_H_10_) attached to ethylamine (C_2_H_7_N)
Karabacak et al. [72]	1	17	8	7	Planar benzene ring with attached B(OH)_2_ and two F groups
Krishnakumar et al. [74]	2	16	4	6	Agrochemical intermediate compounds with planar rings
Krishnakumar et al. [73]	2	16	7	7	Nitrotoluene derivatives
Krishnakumar et al. [75]	2	17	6	7	Phenol derivatives
Kwan and Liu [45]	1	42	22	18	Natural product
Li et al. [76]	78	11	4	5	Molecules with a large number of connected substituent groups
Lomas [77]	15	18	12	5	Saturated alcohols
Osmialowski et al. [78]	28	28	11	14	Substituted phenacylpyridines (ketimine forms) and tautomers
Parlak et al. [79]	1	26	4	12	Polyfluoroaromatic compound with two rings
Perez et al. [80]	2	22	10	8	Chloropyrimidine species
Rablen et al. [81]	80	6	4	11	Rigid organic compounds with constrained conformations
Sarotti and Pellegrinet [82, 83]	66	15	8	6	Low polarity compounds with constrained conformations
Sebestian et al. [84]	1	26	11	14	Phenyl cyanide compound with two planar phenyl rings
Seca et al. [52]	4	40	24	65	Light petroleum extracts
Senyel et al. [85]	1	23	13	9	A structural element of many pharmaceutical drugs
Senyel et al. [86]	1	28	18	8	3-Piperidino-propylamine molecule
Sridevi et al. [87]	1	21	8	10	Chromene, a two ringed planar compound
Tormena and da Silva [88]	3	17	8	8	Para-X-sub’ed (X=H, CH_3_O, & NO_2_) aromatic carbonyl compounds
Vijaja and Sankaran [89]	1	47	24	20	Azine
Watts et al. [53]	6	45	21	18	Coniferol, a building block of lignin, stereoisomers and conformers
Wiitala et al. [90]	43	11	6	3	Organic compounds
Wiitala et al. [91]	7	24	14	8	*Cis*-/*trans*-forms of 2-, 3-, and 4-methylcyclohexanols
Willoughby et al. [92]	2	22	14	7	*Cis*-*/trans*-diastereomers of 3-methylcyclohexanol
Yang et al. [93]	2	57	28	23	Complex natural products
This study’s demonstration set	312	20	10	8	Small- to medium-sized organic molecules with constrained conformations

Details (i.e. experimental conditions, total and average number of nuclei, molecule types and classes) for each molecule set are given in Additional file 1 (S3)

### Computational details

As a first demonstration of ISiCLE, a benchmark study was performed with 8 different DFT methods to predict ^1^H and ^13^C NMR chemical factors for the calculations of chemical shifts in chloroform. Each compound was optimized with the Becke three-parameter Lee–Yang–Parr (B3LYP) hybrid functional [94–96] and the 6-31G(d) split-valence basis set [97]. This level of theory in geometry optimization was chosen because of its broad application in the literature for organic molecules [98, 99]. Isotropic magnetic shielding constants were calculated with the 4 different functionals, BLYP [94, 95], B3LYP [97–99], B35LYP, and BHLYP [100]. DFT methods were selected with different Hartree–Fock (HF) ratios: BLYP (0% HF), B3LYP (20% HF), B35LYP (35% HF), BHLYP (50% HF). Each method was tested with 2 different correlation-consistent Dunning basis sets (double-zeta cc-pVDZ [101] or triple-zeta cc-pVTZ [101]). All basis sets were obtained from the Environmental Molecular Sciences Laboratory (EMSL) Basis Set Exchange [102–104]. For each optimized geometry, ^1^H and ^13^C NMR chemical shifts were computed relative to TMS using the Gauge Including Atomic Orbitals (GIAO) formalism [105]. Chloroform solvation effects were simulated using COSMO.

For a second demonstration of ISiCLE, the NMR chemical shifts, along with frequency calculations (and subsequent Boltzmann weighting), two sets of axial and equatorial conformers (40 conformers each) of methylcyclohexane were processed. We performed *in vacuo* molecular dynamics (MD) simulations, using the sander MD software program from AmberTools (version 14) [106], to generate 80 conformers of the methylcyclohexane compound. These conformers were generated in four stages. First, the initial geometries of axial and equatorial conformers were taken from the study of Willoughby et al. [92]. Second, a short energy minimization run was performed to relax the initial structure and to remove any non-physical atom contacts. Third, a short 50 ps MD run was performed (in 0.5 fs time steps) to heat the structure from 0 to 300 K, without non-bonded cutoffs. In the fourth step, we performed 8 simulated annealing cycles, where each cycle was run for 1600 ps in 1 fs MD steps with the following temperature profile: heating from 300 to 600 K (0–300 ps), equilibration at 600 K (300–800 ps), cooling from 600 to 300 K (800–1100 ps), and equilibration at 300 K (1100–1600 ps). Ten conformers from the equilibration stage at 300 K, of each simulated annealing cycle, were randomly selected to obtain the 80 conformers. After the conformers were obtained, M06-2X was used with the basis set of 6-31 + G(d,p) for the geometry optimization and frequency calculations and B3LYP with 6-311 + G(2d,p) method for the calculations of NMR chemical shifts. Relative free energies of the conformations and Boltzmann weighted NMR chemical shifts were compared to those found in the literature [92, 107–109].

All results shown in this manuscript were generated using the Cascade high-performance computer (1440 compute nodes, 23,040 Intel Xeon E5-2670 processor cores, 195,840 Intel Xeon Phi 5110P coprocessor cores, and 128 GB memory per compute node [110]), in EMSL (a U.S. national scientific user facility) located at PNNL. Cascade is available for external users through a free, competitive proposal process. ISiCLE can utilize local clusters or high-performance computing resources available to the user. NWChem is freely available and can be downloaded from the website [111, 112].

## Results and discussion

NMR chemical shift calculations have been used successfully to identify new molecules, determine metabolite identifications, and eliminate structural misassignments [59, 113]. In the last two decades, many research groups have performed benchmark DFT studies on the accuracy of optimized molecular geometry [92, 114–116], functionals [117, 118], basis sets [88, 119], and solvation models [90, 120, 121] for NMR chemical shifts [60, 122–124]. Each group uses a molecule set focusing on a unique chemical class [78, 125–129] and several groups have recommended different exchange–correlation (XC) energy functionals with a different basis set for a particular condition or suitable to specific chemical functionalities and properties [70, 77, 130–133]. The prevailing opinion is that reliable isotropic NMR chemical shifts strongly depend on accurate calculations of molecular geometries and inclusion of HF exchange in selected DFT methods, to an extent [134, 135]. On the other hand, the size of the basis set does not increase the accuracy after a point [136, 137].

The ISiCLE software can be installed locally. As seen in Fig. 1, it requires only two input files, prepared in Excel: a sequence of InChI or XYZ molecule geometry files, and a sequence of DFT methods of the user’s choice. Preparation of NWChem “run files,” 3D molecule geometry files, and/or Linux/Unix shell script “drivers” are not required. As output, ISiCLE prints isotropic shielding, calculated by NWChem, and calculated chemical shifts with respect to a reference molecule and/or application of a user-specified linear regression technique. ISiCLE is a promising tool contributing to standards-free metabolomics, which depends on the ability to calculate properties for thousands of molecules and their associated conformers.

### Application 1: chemical shift calculations for a demonstration set of molecules

To test ISiCLE, we generated a set of 312 molecules. This set is large relative to other metabolomic molecule sets found in the literature, which in our literature survey averaged 34 molecules (Table 1). Our molecule set ranges from small- to large-sized molecules (number of carbon atoms ranging from 1 to 90), and experimental ^13^C and ^1^H NMR data in chloroform were available for each of them. Our set also spans a wide array of chemical classes, including acetylides, alkaloids, benzenoids, hydrocarbons, lipids, organohalogens, and organic nitrogen and oxygen compounds. ISiCLE was used to successfully perform DFT calculations for this set under chloroform solvation using eight different levels of DFT theory (4 different functionals and 2 basis sets for ^13^C and ^1^H).

**Fig. 3:**
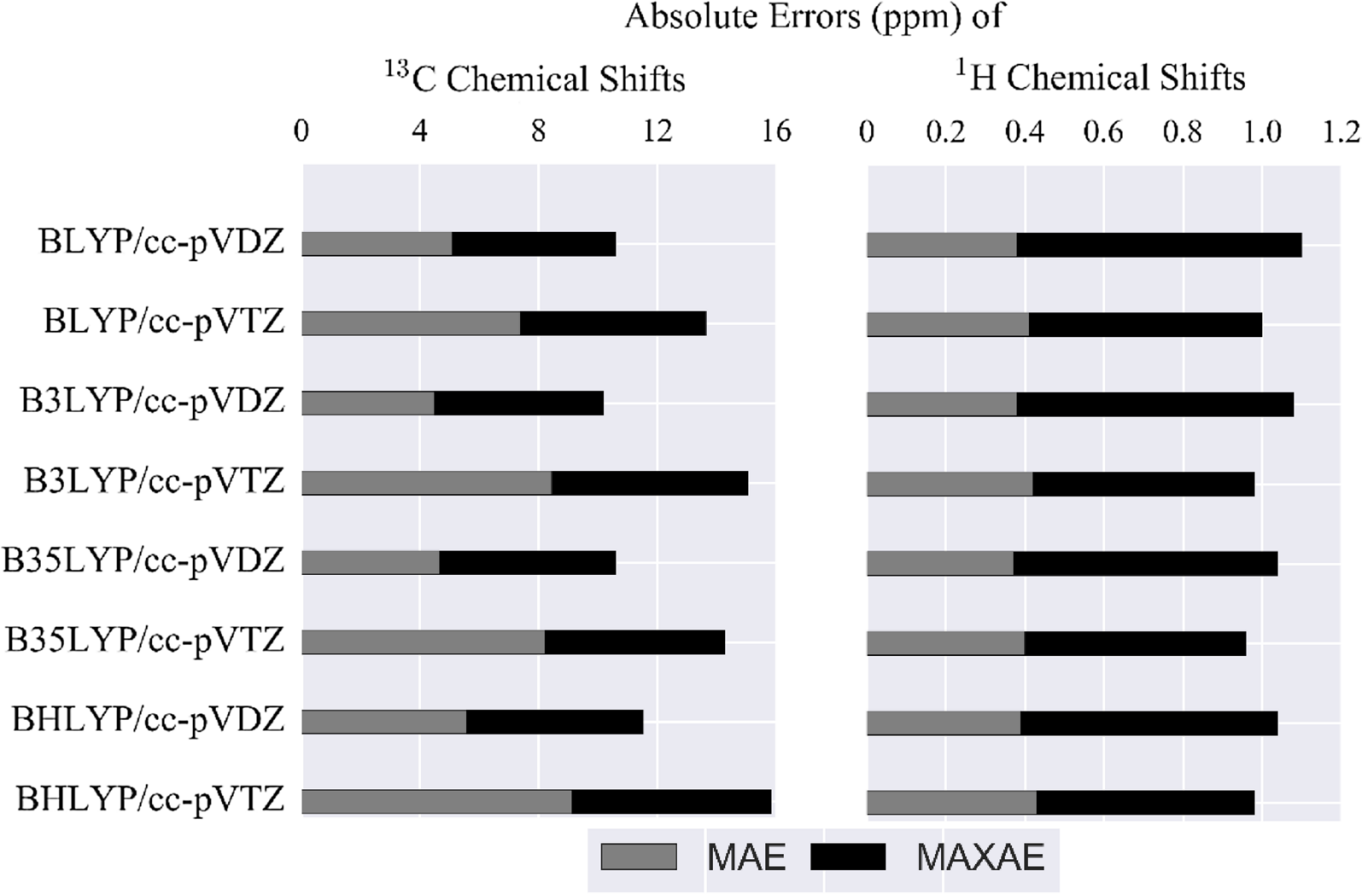
Mean absolute errors (MAE) and maximum absolute errors (MAXAE) of chemical shifts for the demonstration set. The grey bars represent MAE, the black bars represent MAXAE. For all methods, geometries are optimized at B3LYP/6-31G(d) in chloroform

A total of 2494 carbon nuclei and of 3127 hydrogen nuclei were calculated for all 312 molecules of the demonstration set and compared with experimental data. Deviation bars indicating MAE and MAXAE are plotted for each method in Fig. 3. For both ^13^C and ^1^H NMR chemical shifts, the MAE of each method with cc-pVTZ is higher than those with cc-pVDZ. For ^13^C, the MAE of each method with cc-pVTZ (7–10 ppm) is higher than those with cc-pVDZ (5–6 ppm). MAE of methods with a larger basis set deviate more compared to those with a smaller basis set. The smallest deviations are observed for B3LYP and B35LYP, both in MAE and MAXAE results. The same situation is observed for ^1^H NMR chemical shifts as well: MAE of each method with cc-pVTZ (~ 0.35 ppm) is higher than those with cc-pVDZ (~ 0.30 ppm). In contrast to ^13^C NMR chemical shifts, ^1^H NMR chemical shifts are better predicted with methods using larger basis sets (cc-pVTZ). Although the error differences among each method may be too low to confidently identify the outperforming method, B3LYP/cc-pVDZ is the most successful combination in the calculation of ^13^C and ^1^H NMR chemical shifts for our application shown here.

**Fig. 4:**
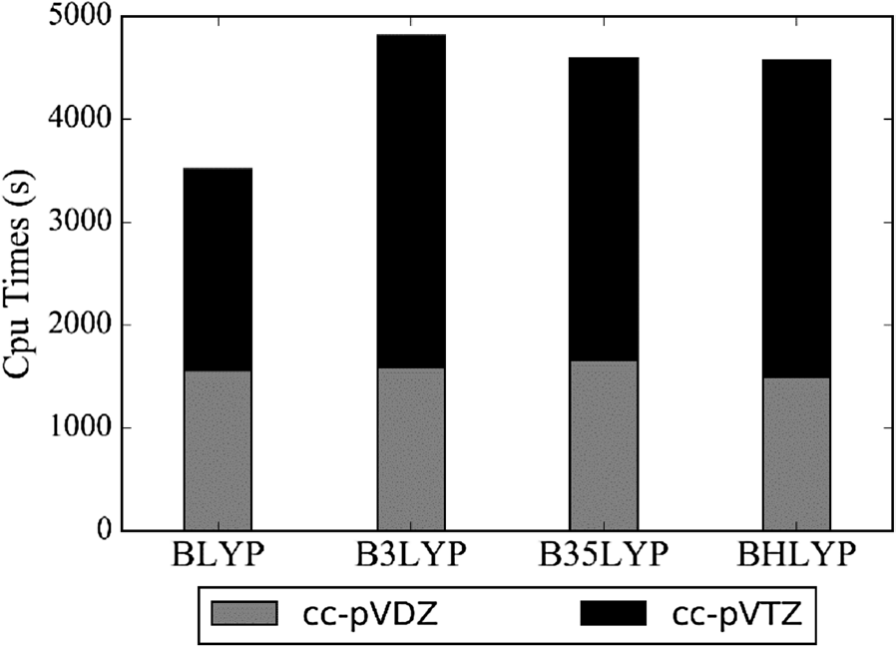
Computational costs of DFT methods performed for the demonstration set. Each bar is for two DFT methods with basis sets of cc-pVDZ and cc-pVTZ. The grey bars represent CPU times for the methods with cc-pVDZ and the black bars represent those with cc-pVDZ and the black bars represent those with cc-pVTZ

Figure 4 shows computational costs of DFT combinations for the demonstration set. We found that the smaller basis set (cc-pVDZ) in the calculation of both ^13^C and ^1^H NMR chemical shifts was an acceptable compromise between accuracy and computational performance, compared with the larger cc-pVTZ basis. This finding is similar to a recent benchmark study [138] that showed B3LYP/cc-pVDZ is a reliable combination, balancing accuracy with computational cost in ^13^C chemical shifts calculation. The larger basis set (cc-pVTZ) took 2–3 times longer to complete than cc-pVDZ (in terms of total CPU time). The computational times of the isotropic shielding and chemical shift calculations for this demonstration set are given in the file of DemonstrationSet_CPUtimes.xlsx in the Additional file 2.

### Effect of scaling by linear regression

**Fig. 5:**
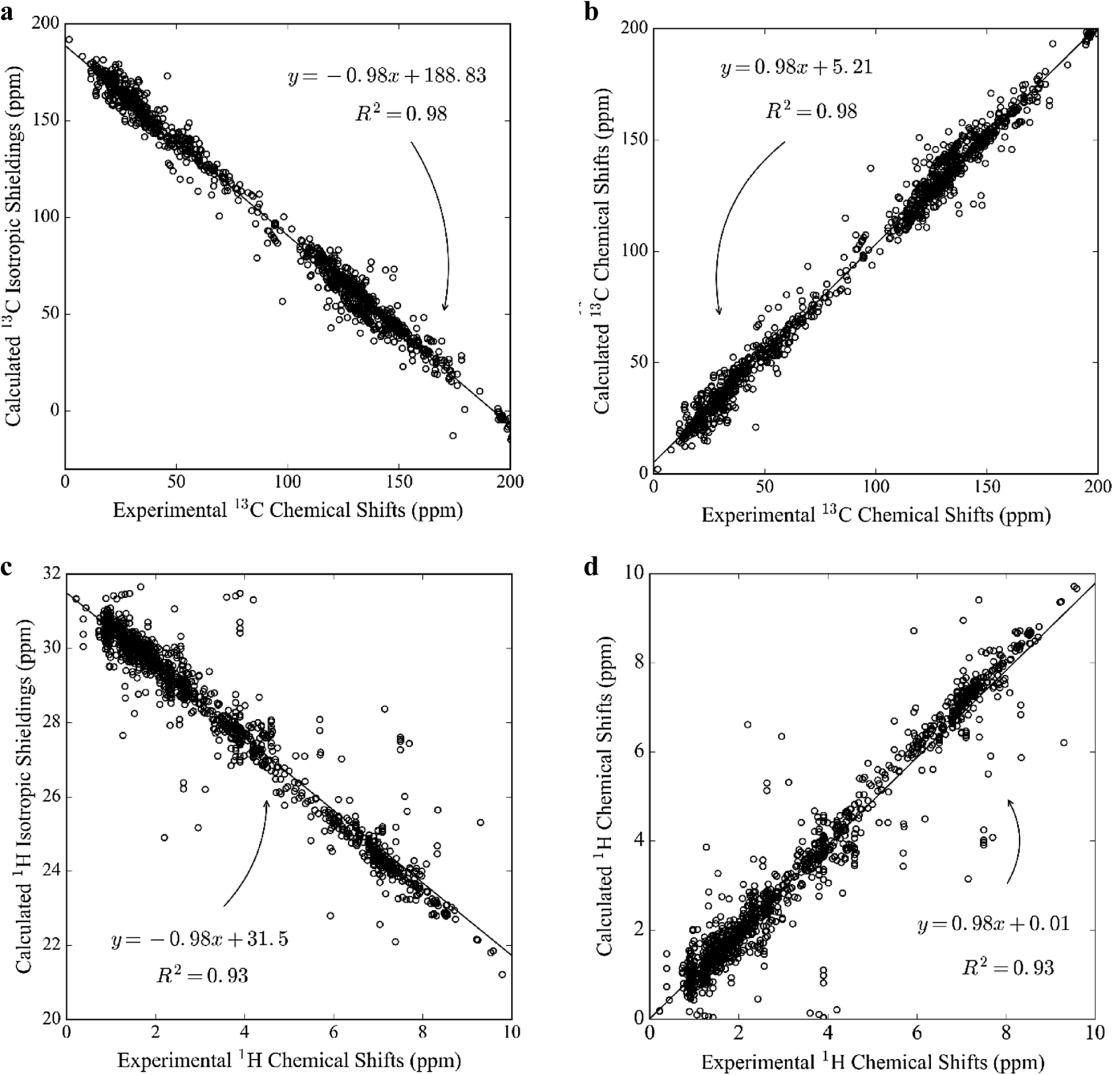
Linear correlation plots of **a** ^13^C and **c** ^1^H isotropic shielding values, and **b** ^13^C and **d** ^1^H NMR chemical shifts versus experimental NMR chemical shifts. Chemical shifts are calculated using the GIAO/B3LYP/cc-pVDZ//B3LYP/6-31G(d) level of theory for the demonstration set in CDCl_3_ (312 molecules (1554 carbons and 1830 hydrogens)). R^2^ indicates the correlation coefficient

We performed the most general approach to error reduction, empirical scaling. Our molecule set has 1554 and 1830 experimental ^13^C and ^1^H NMR chemical shifts, respectively. It provides confidence for applying linear regression effectively as it reduces the possibility of overfitting. Empirical scaling was applied to the data obtained with the best combination, B3LYP/cc-pVDZ, using two different relationships: computed shifts versus experimental chemical shifts (Eq. 6), and computed isotropic shieldings versus experimental shifts (Eq. 7). Once the empirical scaling was applied, the accuracy for ^13^C chemical shifts and ^1^H chemical shifts improved by 0.7 and 0.11 ppm, respectively. Our computed NMR chemical shifts and shieldings deviate from unity (desired slope = 1) by 0.02 for both ^13^C and ^1^H NMR chemical shifts. Linear fits with correlation coefficients of 0.99 (Fig. 5a, b) and 0.93 (Fig. 5c, d) for ^13^C and ^1^H NMR chemical shifts, respectively, were observed, which also shows that B3LYP/cc-pVDZ is able to produce data free from random error. Results of linear regression to the ^13^C and ^1^H NMR chemical shifts obtained by other DFT methods are given in the Additional file 1 (S5).

### Detailed look at ^13^C NMR chemical shifts

The carbon (^13^C) magnetic shieldings and chemical shifts derived from the various DFT methods are highly correlated, as shown by a correlation coefficient of 0.99 (Fig. 5). The inclusion of a scaling factor enhances the performance of theoretical calculations with B3LYP/cc-pVDZ//B3LYP/6-31G(d) and decreases the MAE in ^13^C NMR chemical shifts for this set by approximately 13%.

**Fig. 6:**
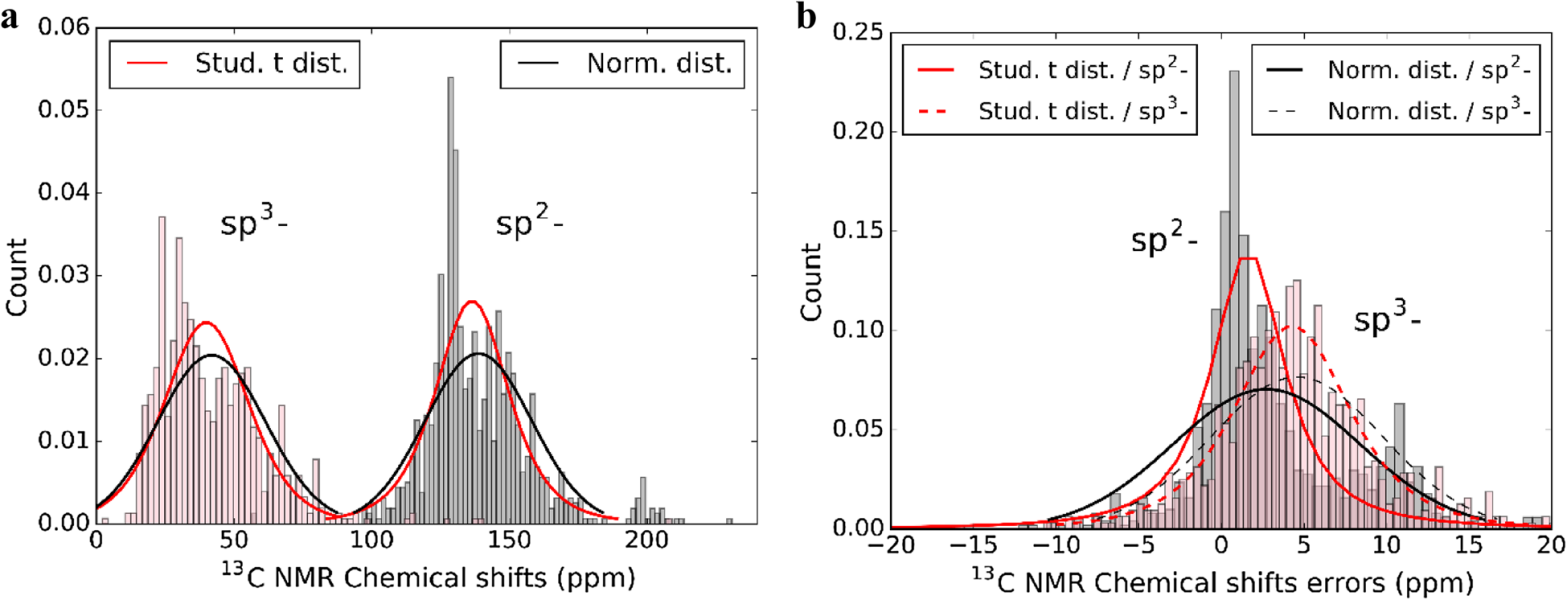
Chemical shifts of sp2- and sp3- hybridized carbon atoms. **a** Chemical shifts, **b** associated errors. Chemical shifts were calculated using the B3LYP/cc-pVDZ//B3LYP/6-31G(d) level of theory in CDCl_3_

There has been a trend toward using multiple references, such that each molecule should be referenced to a molecule with similar properties to improve accuracy of NMR chemical shifts [123, 134, 139]. Sarotti et al. examined the influence of the reference compound used in the ^13^C [83] and ^1^H [82] NMR chemical shift calculations over a set of organic compounds, all of which were included in our calculations. They recommended the use of benzene and methanol as a reference standard in the calculations of chemical shifts of sp-sp^2^- and sp^3^- hybridized carbon atoms, respectively, instead of TMS for all type of carbon atoms [140]. Propelled by the discussion in the study of Grimblat et al. [141] about the distribution of the errors observed in sp^2^- and sp^3^- carbons, we determined the distribution of the data of chemical shifts of sp^2^- (933 carbons) and sp^3^- (745 carbons) hybridized carbons (Fig. 6, the sp^2^- and sp^3^- derived series of carbons show two separate chemical shift distributions and two separate error distributions over a much larger variety of compounds than Grimblat et al. For our demonstration set, both errors between calculated and experimental sp^2^- and sp^3^- chemical shifts more closely resemble a Student’s t-distribution [58, 142], rather than a normal distribution [138, 143]. The correlation coefficients of the errors of sp^2^- and sp^3^- carbons are 0.93 and 0.78, and 0.98 and 0.95 for Student’s t-distribution and normal distribution, respectively.

**Fig. 7:**
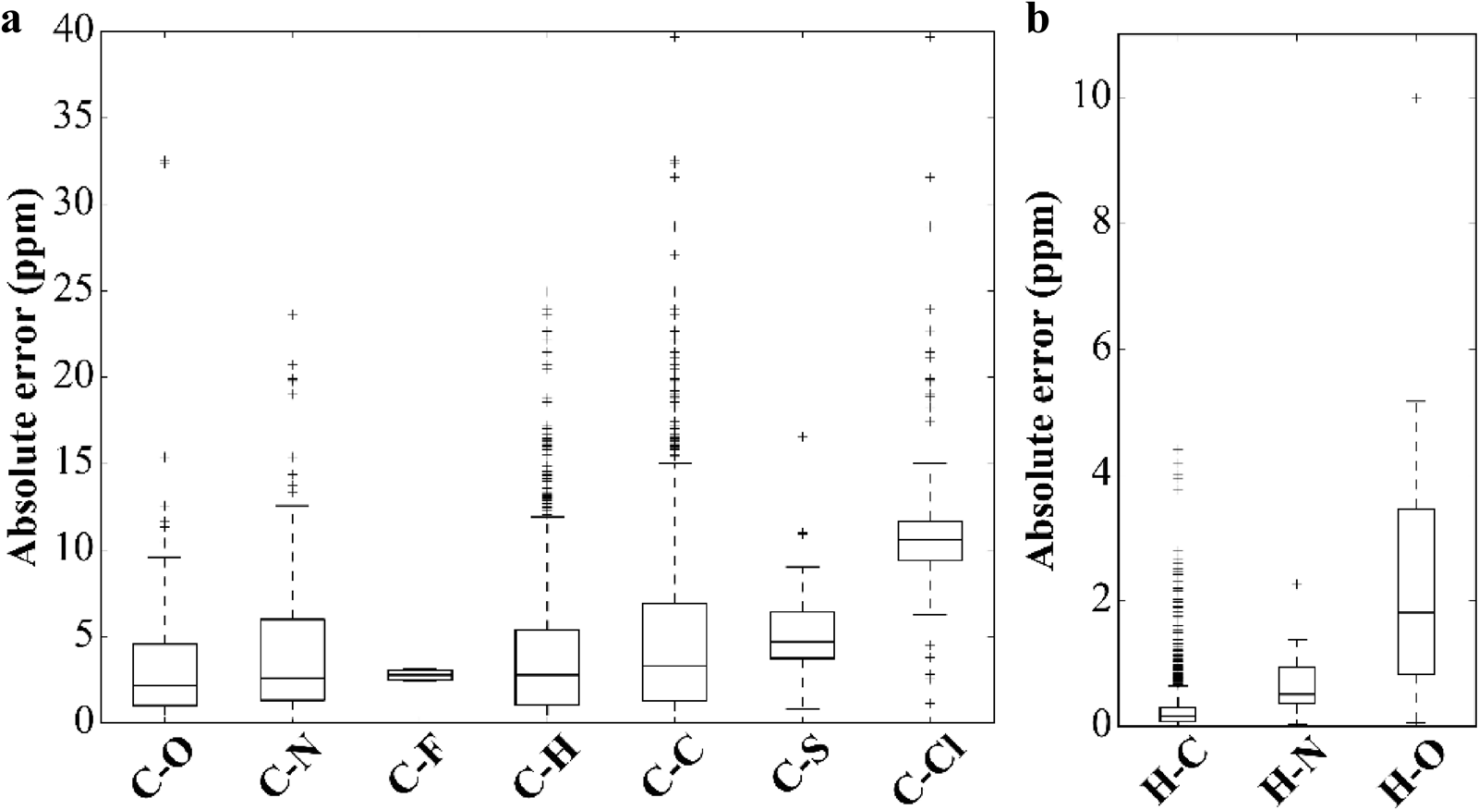
Chemical shift prediction errors for different functional groups. **a** ^13^C NMR chemical shifts, **b** ^1^H NMR chemical shifts. All molecules are from the demonstration set and are calculated using the GIAO/B3LYP/cc-pVDZ//B3LYP/6-31G(d) level of theory in chloroform

Furthermore, we looked for the bonded neighbors of each carbon and hydrogen extensively in Fig. 7. For carbon shifts, error was measured for carbon (n = 1709), chlorine (n = 149), fluorine (n = 8), hydrogen (n = 1161), nitrogen (n = 199), oxygen (n = 251) and sulfur (n = 20) attachments. The largest deviations occur in carbon–chlorine and carbon–sulfur attachments with MAEs of 11.2 and 5.8 ppm and MAXAE 39.7 and 16.5 ppm, respectively. The study by Li et al. [76], which used a set of chlorinated carbons, reports the same conclusion: calculation accuracy decreases as the size of the basis set used increases, but improvement was obtained after linear regression corrections for B3LYP/6-31 + G(d,p) with slope of 0.98. Other than chlorine and sulfur, carbon-hydrogen attachments also make the ^13^C NMR chemical shift DFT calculations deviate significantly from experimental values, with MAE of 4.7 and 3.9 ppm and with MAXAE of 51.6 and 34.9 ppm. Carbon was found in rings in 70% of the cases, and these carbons show a MAE of 4.6 ppm. Also, the MAE of ^13^C NMR chemical shift is 3.9 ppm for carbons bonded to a hydrogen atom but reaches 9.7 ppm in all other cases.

Oxygen and nitrogen attachments to carbon led to ^13^C NMR chemical shifts with MAE up to 3.1 and 4.2 ppm and MAXAE of 32.5 and 23.6 ppm, respectively. Interestingly, half the C–O attachments found in ring-form had chemical shifts with a MAE of 2.8 ppm, compared to the chemical shifts of C–O attachments not found in a ring, which had a relatively higher MAE of 3.2 ppm, leading to a percent difference of 14.1%. NMR chemical shifts of C–N attachments, present in a ring or not, show close MAE of 4.19 and 4.26 ppm, respectively, a percent difference of 1.6%. This is to be expected, since C–O attachments are expected to show some deviation in chemical shift due to the polarization of the electron distribution caused by the high electronegativity of oxygen, while nitrogen atoms have a lower electronegativity, leading to a lower deviation in C–N chemical shifts.

The correlation plot (Fig. 5a, b) shows a linear pattern with only minor deviations of the predicted ^13^C shieldings or ^13^C chemical shifts from the fitted line. It is verified by the correlation coefficient of 0.99, as observed in previous studies [130, 144], that the deviation of the slope from unity within the range of 0.95 and 1.05 is an indicator of a reliable method. However, when placed in subgroups of different attachment types, distant outliers are observed, with some more than 15 ppm away (Fig. 7a). Most outliers are observed in the C–C and C–H attachments, respectively. C–Cl and C–N chemical shifts have a high occurrence of outliers, which may be due to the chemical properties of chlorine and nitrogen such as being remarkably close to first ionization energies. We suspected that some cases of high calculation errors could be due to the consideration of only a single conformer. For the highest accuracy, proper conformational sampling must be considered, as demonstrated below in “Application 2: Boltzmann-weighted NMR chemical shifts of methylcyclohexane” section

### Detailed look at ^1^H NMR chemical shifts

Proton (^1^H) chemical shifts are significantly affected by intermolecular interactions, particularly in aqueous states, especially compared to ^13^C chemical shifts. Agreement with experimental values improves as empirical linear scaling is performed for ^1^H chemical shifts. GIAO/B3LYP/cc-pVDZ//B3LYP/6-31G(d) yields scaled ^1^H chemical shifts in chloroform solution having a MAE of 0.30 ppm in comparison with solution experimental values. The ^1^H chemical shifts in the range of 10–17 ppm show the largest deviation, occurring higher than 5 ppm.

In Fig. 7b, error bars are shown for ^1^H chemical shifts when the hydrogen attaches to carbon (n = 1793), nitrogen (n = 17), and oxygen (n = 41). Oxygen-bound hydrogen nuclei have the largest errors (up to 10 ppm), which is to be expected due to the electronegative property of oxygen atoms, as discussed in the previous section. It is followed by less electronegative nitrogen-bound hydrogen atoms, with an MAE of 0.71 ppm and a MAXAE of 2.25 ppm. About 95% of the ^1^H NMR chemical shifts calculated for this set are from H–C attachments. These chemical shifts had a MAE of 0.27 ppm and a MAXAE of 4.41 ppm. The high occurrence of outliers could be evidence of how ^1^H NMR chemical shifts are sensitive to intermolecular interactions.

H–O attachments are highly sensitive (to concentration, solvent, temperature, etc.), and it is non-trivial to determine the NMR chemical shift value of arbitrary protons experimentally as well as predict them by using a single, “catch all” DFT method, which explains the relatively low correlation coefficient of 0.93 (Fig. 5c–d). For future studies, we may need to consider the use of different DFT methods, including the use of explicit solvation, particularly in the calculation of ^1^H NMR chemical shifts in the presence of H–O attachments.

Application of empirical scaling to functional groups are given in detail in the Additional file 1 (see S7 and S8).

### Cross-validation

As a final assessment for the data collected with the demonstration set, we assessed the stability and accuracy of the linear regression approach using cross-validation [145]. Cross-validation is a technique mostly used in prediction problems to evaluate how much a given model generalizes to an independent set of data. Specifically, we performed Monte Carlo cross-validation [146, 147]. The procedure of application of Monte Carlo cross-validation method is explained in Additional file 1 (S9).

We observed that the estimated linear model parameters (i.e. slope and intercept) from the training set do not differ from that of the entire set. Therefore, the predictive linear model is stable to be accurately estimated and the subsets of ^13^C and ^1^H NMR chemical shifts generalize well to the groups that are not represented in the training fold.

### Application 2: Boltzmann-weighted NMR chemical shifts of methylcyclohexane

Metabolites were experimentally interrogated using solution-state NMR, where the observed signal arises from the combined signals of present conformers. It is routine that NMR chemical shifts calculations are carried out on a single dominant conformer. However, it is well known that metabolites do not comprise a single conformer in solution and are instead found in a collection of various conformers [148], and the accuracy of NMR chemical shifts heavily depends on molecular geometries and conformation consideration [46]. It has been shown that for the highest accuracy NMR chemical shift calculations, consideration of conformers is critical, even for relatively small molecules [149]. As a second demonstration of ISiCLE, a conformational analysis based on DFT was performed on a set of 80 conformers of methylcyclohexane using a Boltzmann distribution technique. Boltzmann weighting determines the fractional population of each conformer based on its energy level [92]. High-throughput and straightforward DFT-based NMR chemical shift calculations of all 80 methylcyclohexane conformers was performed by ISiCLE then compared to experimental values.

**Fig. 8:**
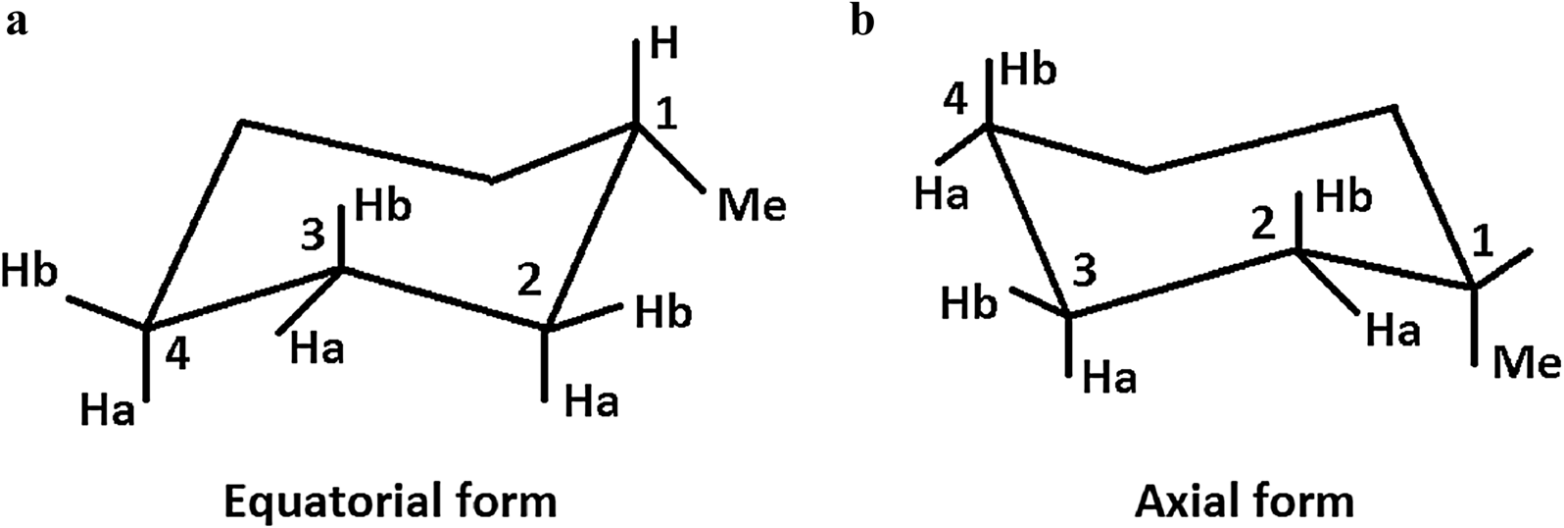
The **a** equatorial and **b** axial structures of methylcyclohexane

**Fig. 9:**
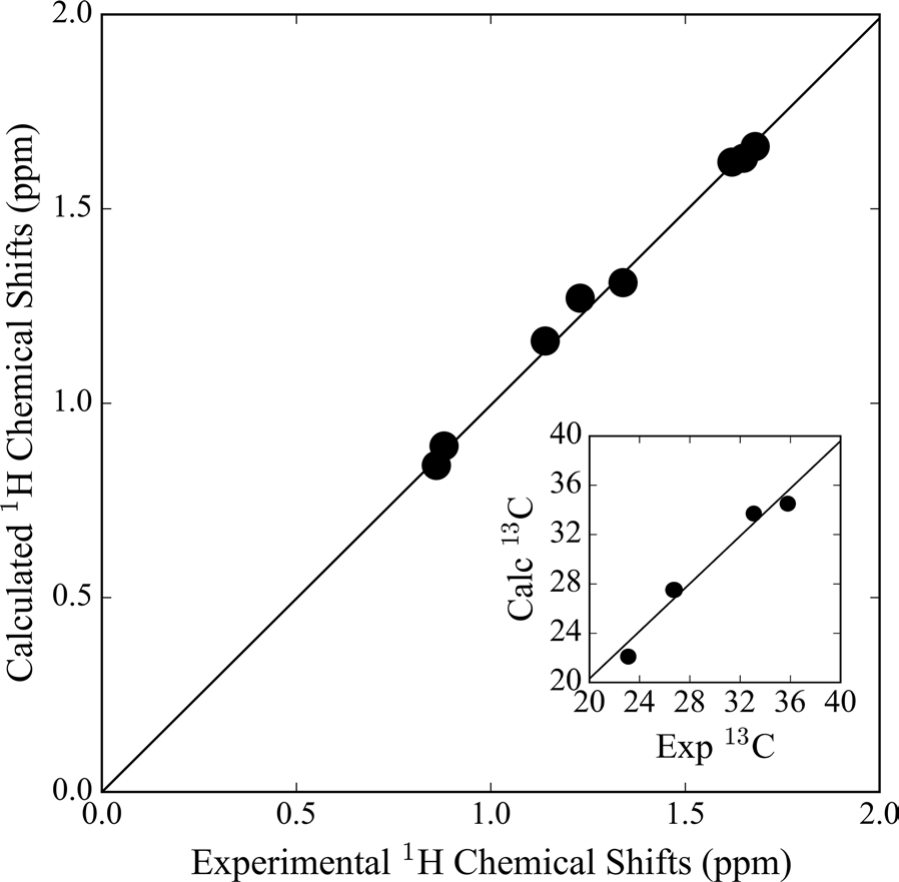
Experimental and scaled chemical shifts (ppm) of methylcyclohexane

It has been shown by Willoughby et al. [92] that the effects of molecular flexibility on NMR chemical shifts can be captured by Boltzmann weighting analysis, as demonstrated with methylcyclohexane (Fig. 8). Methylcyclohexane is a well-studied small molecule [150–154]. It is flexible, composed of a single methyl group attached to a six-membered ring, and known to exist as an assembly of two chair conformers. There are two distinct conformations of which chair–chair interconversion is rapid and dominated by equatorial to axial conformation. We weighted 40 axial and 40 equatorial conformers in chloroform and obtained a relative free energy of 1.99 kcal/mol with NWChem, similar to calculations using Gaussian [155] by Willoughby et al. [92], and similar to experimental findings (1.73 kcal/mol [156], 1.93 kcal/mol] [108]) and computed (2.15–2.31 kcal/mol [107] and 1.68–2.48 kcal/mol [109]) values. We compared the Boltzmann-weighted ^1^H and ^13^C chemical shifts (a ratio of 3% axial to 97% equatorial) to experimental values reported by Willoughby et al. [92]. The Boltzmann-weighted, scaled MAE was 0.017 ppm for ^1^H chemical shifts ($\delta_{exp} = 1.00 \times \delta_{comp} + \sim0.00$), similar to the experimental value of 0.018 ppm in the study of Willoughby et al. Also, the MAE for ^13^C chemical shifts was 4.4 ppm and decreases to 0.8 ppm when the chemical shifts were scaled ($\delta_{exp} = 0.99 \times \delta_{comp} + 0.13$) (Fig. 9). Further details can be found in the Additional file 1 (S10).

## Conclusions

We introduce the first release of ISiCLE, which predicts NMR chemical shifts of any given set of molecules relevant to metabolomics for a given set of DFT techniques. ISiCLE calculates the unscaled or scaled NMR chemical shifts (depending on the user’s choice of DFT method) and writes the data to appended MDL Molfiles. It also quantifies the error in calculated NMR chemical shifts if the user provides experimental values.

The functionality of ISiCLE is demonstrated on a molecule set consisting of 312 molecules, with experimental chemical shifts reported in chloroform solvent. 1H and 13C NMR chemical shifts were calculated using 8 different levels of DFT (BLYP, B3LYP, B35LYP, BHLYP and, cc-pVDZ, and ccpVTZ), referenced to TMS in chloroform by carrying initial geometry optimizations out at B3LYP/6-31G(d) for all molecules. The optimal combination for this set was found to be B3LYP/cc-pVDZ//B3LYP/6-31G(d) with mean absolute error of 0.33 and 3.93 ppm for proton and carbon chemical shifts, respectively. We show that DFT calculations followed by linear scaling do in fact provide an analytically useful degree of accuracy and reliability. Finally, we used ISiCLE for the calculation of NMR chemical shifts of 80 Boltzmann-weighted conformers of methylcyclohexane and compared our results with earlier studies in the literature.

ISiCLE is a promising automated framework for accurate NMR chemical shift calculations of small organic molecules. Through this tool, we hope to expand chemical shift libraries, without the need for chemical standards run in the laboratory, which could lead to significantly more identifiable metabolites. Future work includes wrapping individual steps of the ISiCLE NMR module into a formal workflow management system such as Snakemake, to include better fault tolerance, modularization, and improved data provenance. Furthermore, additional chemical properties will be included, such as ion mobility collision cross section and infrared spectra. Finally, ISiCLE will be adapted to run seamlessly on cloud computer resources such as Amazon AWS, Microsoft Azure, and Google Cloud. ISiCLE is a promising tool contributing to standards-free metabolomics, which depends on the ability to calculate properties for thousands of molecules and their associated conformers.

## Additional files

**Additional file 1.** Supporting information document.

**Additional file 2.** Supporting information files. Including tutorial, code, and all other files.
