# Supplementary material for: An automated framework for NMR chemical shift calculations of small organic molecules
Source: J Cheminform. 2018 Oct 26;10:52. doi: 10.1186/s13321-018-0305-8 (PMC6755567; doi:10.1186/s13321-018-0305-8)
Supplement: Supplementary file 1 — Additional file 1. Supporting information document. [file 13321_2018_305_MOESM1_ESM.docx]

**Supplementary Information**

**An automated framework for NMR chemical shift calculations of small organic molecules**

**Authors:**

Yasemin Yesiltepe^1,2^, Jamie R. Nuñez^2^, Sean M. Colby^2^, Dennis G. Thomas^2^, Mark I. Borkum^2^, Patrick N. Reardon^3^, Nancy M. Washton^2^, Thomas O. Metz^2^, Justin G. Teeguarden^2^, Niranjan Govind^2^, Ryan S. Renslow^1,2^*

^1^ The Gene and Linda Voiland School of Chemical Engineering and Bioengineering, Washington State University, Pullman, WA, USA.

^2^ Earth and Biological Science Division, Pacific Northwest National Laboratory, Richland, WA, USA.

^3^ Nuclear Magnetic Resonance Facility, Oregon State University, Corvallis, OR, 97331, USA

*Corresponding author: Ryan Renslow ([ryan.renslow@pnnl.gov](mailto:ryan.renslow@pnnl.gov))

**Contents**

[Figures 3](#_Toc515804116)

[Tables 4](#_Toc515804117)

[S1. ISiCLE 5](#_Toc515804118)

[S2. InChI, InChIKey and Molfiles 7](#_Toc515804119)

[S3. Demonstration Set 10](#_Toc515804120)

[S4. Error analysis of chemical shift calculations for the demonstration set 15](#_Toc515804121)

[S5. Performance vs. computational cost 16](#_Toc515804122)

[S6. Application of Empirical Scaling Derived from Linear Regression Approach 17](#_Toc515804123)

[S7. Application of Empirical Scaling to Functional Groups 27](#_Toc515804124)

[S8. Tetramethylsilane 29](#_Toc515804125)

[S9. Monte Carlo Cross-validation 31](#_Toc515804126)

[S10. Conformational Analysis of Methylcyclohexane 33](#_Toc515804127)

# **Figures**

[Figure 1. Mean absolute errors (MAE) and maximum absolute errors (MAXAE) of chemical shifts for the demonstration set. 14](#_Toc515805580)

[Figure 2 Computational costs of DFT methods performed for the demonstration set. 15](#_Toc515805581)

[Figure 3. Linear correlation plots of ^13^C and ^1^H NMR chemical shifts calculated using the DFT method: GIAO/BLYP/cc-pVDZ//B3LYP/6-31G(d) for the demonstration set in CHCl_3_. 18](#_Toc515805582)

[Figure 4. Linear correlation plots of ^13^C and ^1^H NMR chemical shifts calculated using the DFT method: GIAO/BLYP/cc-pVTZ//B3LYP/6-31G(d) for the demonstration set in CHCl_3_. 19](#_Toc515805583)

[Figure 5. Linear correlation plots of ^13^C and ^1^H NMR chemical shifts calculated using the DFT method: GIAO/B3LYP/cc-pVDZ//B3LYP/6-31G(d) for the demonstration set in CHCl_3_. 20](#_Toc515805584)

[Figure 6. Linear correlation plots of ^13^C and ^1^H NMR chemical shifts calculated using the DFT method: GIAO/B3LYP/cc-pVTZ//B3LYP/6-31G(d) for the demonstration set in CHCl_3_. 21](#_Toc515805585)

[Figure 7. Linear correlation plots of ^13^C and ^1^H NMR chemical shifts calculated using the DFT method: GIAO/B35LYP/cc-pVDZ//B3LYP/6-31G(d) for the demonstration set in CHCl_3_. 22](#_Toc515805586)

[Figure 8. Linear correlation plots of ^13^C and ^1^H NMR chemical shifts calculated using the DFT method: GIAO/B35LYP/cc-pVTZ//B3LYP/6-31G(d) for the demonstration set inCHCl_3_. 23](#_Toc515805587)

[Figure 9. Linear correlation plots of ^13^C and ^1^H NMR chemical shifts calculated using the DFT method: GIAO/BHLYP/cc-pVDZ//B3LYP/6-31G(d) for the demonstration set in CHCl_3_. 24](#_Toc515805588)

[Figure 10. Linear correlation plots of ^13^C and ^1^H NMR chemical shifts calculated using the DFT method: GIAO/BHLYP/cc-pVTZ//B3LYP/6-31G(d) for the demonstration set in CHCl_3_. 25](#_Toc515805589)

[Figure 11. Monte Carlo cross-validation results. 31](#_Toc515805590)

[Figure 12. Linear correlation plot of experimental and Boltzmann-weighted ^1^H NMR chemical shifts (ppm) calculated at GIAO/ B3LYP/6-311+G(2d,p)//M06-2X/6-31+G(d,p) level of theory 37](#_Toc515805591)

[Figure 13. Linear correlation plot of experimental (taken from Ref [69] (black) and Ref [68] (red)) and Boltzmann-weighted ^13^C NMR chemical shifts (ppm) calculated at GIAO/ B3LYP/6-311+G(2d,p)//M06-2X/6-31+G(d,p) level of theory. 39](#_Toc515805592)

# **Tables**

[Table 1. Demonstration set sources and details. 11](#_Toc514590133)

[Table 2. Average results of unscaled and scaled ^13^C chemical shifts for each DFT method. 18](#_Toc514590134)

[Table 3. Average results of unscaled and scaled ^1^H chemical shifts for each DFT method. 18](#_Toc514590135)

[Table 4. Linear regression parameters for subgroups of carbon and hydrogen nuclei 24](#_Toc514590136)

[Table 5. Linear regression parameters for carbon nuclei with excluded nucleus 24](#_Toc514590137)

[Table 6. Linear regression parameters for hydrogen nuclei with excluded nucleus 25](#_Toc514590138)

[Table 7. Linear regression parameters for the plots f experimental shifts vs. shielding tensors for carbon and hydrogen. 26](#_Toc514590139)

[Table 8. Initial geometry of axial methylcyclohexane [52] 30](#_Toc514590140)

[Table 9. Initial geometry of equatorial methylcyclohexane [52] 31](#_Toc514590141)

[Table 10. NMR shielding tensors of axial and equatorial methylcyclohexane 32](#_Toc514590142)

[Table 11. ^1^H computed NMR chemical shifts of axial and equatorial methylcyclohexane 33](#_Toc514590143)

[Table 12. Experimental and Boltzmann-weighted and scaled ^1^H chemical shifts (ppm) of methylcyclohexane calculated at B3LYP/6-311+G(2d,p)//M06-2X/6-31+G(d,p). I: Absolute deviation (ppm) in Boltzmann weighted ^1^H chemical shifts. II: Absolute deviation (ppm) reported [52]. III: Absolute deviation (ppm) in scaled ^1^H chemical shifts. 34](#_Toc514590144)

[Table 13. Experimental and calculated at B3LYP/6-311+G(2d,p)//M06-2X/6-31+G(d,p) (ppm) of methylcyclohexane. 35](#_Toc514590145)

[Table 14. Boltzmann-weighted and scaled ^13^C chemical shifts (ppm) of methylcyclohexane. I: Absolute deviation (ppm) in Boltzmann weighted ^13^C chemical shifts. II: Absolute deviation (ppm) in scaled ^13^C chemical shifts. 36](#_Toc514590146)

# **S1. ISiCLE**

The *in silico* Chemical Library Engine (ISiCLE)’s NMR module is a software package that automates NMR chemical shift calculation. It’s user-friendly, written in Python, and can be installed on a personal computer. All DFT calculations are performed with NWChem, an open-source, high-performance computational chemistry codebase developed at Pacific Northwest National Laboratory (PNNL). ISiCLE is able to interact with remote installations of NWChem on supercomputing resources.

NWChem accepts text files as input, containing job title, start-up directives that define the general features of the calculation—including available memory, name of the database, and locations for where to save scratch/permanent files —and other global options. The configuration file also contains the definition of the geometry file and indication of which DFT methods will be used for optimization. NWChem input files are prepared automatically by interfacing with a user-supplied Excel spreadsheet.

Moreover, NWChem supports a number of DFT theory levels, and users may specify their own NWChem input files for specific functions, such as loading of the geometry explicitly, contributing to a local functional, specifying angular grids, etc. Details about the required format is further discussed in the NWChem user manual or NWChem webpage.

ISiCLE can be run locally after installing OpenBabel, an open source software that is available for Windows, Linux, and macOS. In addition, ISiCLE can interface with remote computing resources through Fabric [[1](#_ENREF_1)], a Python-based command line tool for streamlining the use of secure shell (SSH). Fabric is used to execute remote shell commands, upload and download files or folders, and so on.

Once input files are generated locally, ISiCLE uploads them to a remote supercomputing resource and submits to the associated scheduling system. Once the simulation starts, ISiCLE notifies the user of the job status and provides a list of the molecules whose simulations have been launched. Then, it tracks the progress of the simulation, remaining time, and completed molecules.

Sometimes simulation of a molecule can fail due to an error related to input files, geometry, and/or insufficient time. In the event of failure, ISiCLE resubmits up to three times, requiring the user to input a new time limit. After the given jobs are finished, ISiCLE checks if the files have a character reserved in the Windows operating system or not (i.e. characters such as “/” (forward slash), “\” (backslash), * (asterisk) etc.). If any, it replaces them with their wordings. Subsequently, it transfers all files to the local machine. Critical files are then checked to make sure they were both uploaded and downloaded correctly to the compared files are identical. A warning message is raised to inform the user of any file transfer errors.

# **S2. InChI, InChIKey and Molfiles**

Thanks to the increase in the availability of public and commercial chemical structure databases, the past decade has seen an increase in researchers involved in the fields of cheminformatics, bioinformatics, systems biology, and translational medicine [[2](#_ENREF_2), [3](#_ENREF_3)]. Particularly, resources such as PubChem [[4](#_ENREF_4)], RSC ChemSpider [[5](#_ENREF_5)], ChEMBL [[6](#_ENREF_6), [7](#_ENREF_7)], ChEBI [[8](#_ENREF_8), [9](#_ENREF_9)], DrugBank [[10](#_ENREF_10), [11](#_ENREF_11)], BMRB [[12](#_ENREF_12)] and HMDB [[13](#_ENREF_13)] have shown great impacts to research community [[2](#_ENREF_2)]. Many new compounds are published in journals and patents every year, and the importance of attributing correct structures and storing in easily accessible databases is mounting [[14](#_ENREF_14), [15](#_ENREF_15)]. The complexity of new molecular structures makes the conventional naming procedures inconvenient and reveals the necessity of suitable, openly available electronic format for linking chemical structures over the internet [[16](#_ENREF_16)]. MDL Molfile [[17](#_ENREF_17)], SMILES notations [[18-20](#_ENREF_18)], IUPAC standardized names [[21](#_ENREF_21)], and InChI identifiers [[22](#_ENREF_22), [23](#_ENREF_23)] are ubiquitous file formats and chemical identifiers. In particular, InChI is a freely available, non-proprietary chemical identifier that can be used in printed and electronic data sources.

ISiCLE uses InChI strings, or a free-format text file having XYZ coordinates of atoms, for each molecule of interest as part of the input. In either case, ISiCLE detects the file type and converts to XYZ if necessary, which is the file format accepted by NWChem.

InChI processing operations rely on OpenBabel, an open-source chemical toolbox, for conversions of given data. OpenBabel supports many types of chemical data and provides extensive capabilities for reading and writing molecular file formats, interconversion of chemical structures between different formats, manipulation of molecular data, etc.

InChI were chosen as the key molecule identifier because they enable easy linking of data compilations and allow for unambiguous identification. Secondly, according to the study of Akhondi et al. [[24](#_ENREF_24)] on the inconsistency of these structure identifiers due to various approaches for data integration, including the use of different software and different rules for structure canonicalization, InChI had the highest consistency within and between commonly used cheminformatics resources, with and without structure canonicalization. It offers the most successful conversion to MDL Molfiles and SMILES. Thirdly, it offers InChIKey, which facilitates web searching, enabling the development of web-based InChI lookup services, permitting an InChI identifier to be stored in fixed length fields, facilitating chemical structure indexing in databases. Last but not least, in the longer term, InChI identifiers will be expanded to cover macromolecules, positional isomers, crystal structures, inorganic, organometallic, coordination compounds, and tautomers [[25](#_ENREF_25)].

InChI is a machine-readable string of characters that enables a computer to represent a chemical structure in a canonical form, such that the original structure can be regenerated from an InChI with appropriate software. While the InChI is a unique string identifying a defined chemical structure, ISiCLE uses the InChIKey, a 27-character hashed version of the InChI identifier, for naming output files. The InChIKey standard is based on the SHA-256 hash of the original InChI identifier. The resulting InChIKey is divided into two blocks. The first block encodes the molecular skeleton, while the second block represents various kinds of isomerism (stereo, tautomeric, etc.).

We have two reasons to prefer InChIKeys for naming files rather than using InChI identifiers directly. Firstly, InChIKey is intended for web and database searching and indexing, especially it is nontrivial for search engines to properly search for a lengthy InChI string [[26](#_ENREF_26), [27](#_ENREF_27)]. Secondly, the length of an InChI identifier increases with the size of the structure. Currently, InChI allows for up to 1000 atoms; but, even a structure with 100 atoms is represented by a very long string. On the other hand, as noted previously, InChIKey is a compacted version of InChI and consists of only 27 characters, regardless of molecule size. Also, Windows operating system has a character limit for file names and prohibits the usage of some characters such as slash which is repeatedly present in InChI code. Lastly, InChIKey will enhance usefulness as a search tool when derived from a “standard” InChI, which is expected to include additional features such as tautomerism and stereochemistry.

Although the InChIKey is intended to be a unique substitute for the parent InChI, a single InChIKey may occasionally map to two or more InChI strings (i.e. a hash collision). Because it is a hash, the InChIKey cannot be converted into the original structure. Collision-free hashing is impossible within a finite hash space, and InChIKeys have a reasonable level of collision resistance, sufficient for typical applications [[28](#_ENREF_28)]. That is why we do not expect to encounter any problems sourcing from the collisions in InChIKey.

InChIs are included in many well-known public and commercial databases such as Thomson Reuters Web of Science, ChEMBL, ChEBI, DrugBank, the US National Cancer Institute, Chemical Family database, etc. They contain millions of InChIs and InChIKeys. We prefer the two very large databases: RSC ChemSpider and PubChem to collect InChI strings for our demonstration set. RSC ChemSpider offers search facilities and web services enabling a variety of InChI and InChIKey conversions. It also allows searching for molecules by simply drawing their structures. PubChem provides InChI-based structure search facilities.

OpenBabel reads each given InChI and creates an initial molecule object with 2D Cartesian coordinates and connectivity information. Subsequently, OpenBabel applies the MMFF94 force field to generate a rough 3D structure of the molecule. MMFF94 is the default force field in OpenBabel, and is frequently used for small organic molecules, but users may select other types of force fields (e.g. GAFF, UFF) as needed. For example, some molecules have elements that may be unsupported by MMFF94. The installation details for OpenBabel and other packages are provided in the tutorial.

ISiCLE also generates 3D MDL Molfiles. MDL Molfiles were selected for two reasons. Firstly, the MDL Molfile format is a very well established data exchange. MDL Molfiles also provides basic fault tolerance: lines not understood by cheminformatics software (e.g. Jmol, Marvin, IQMol, Rasmol) are simply ignored. The supplementary tutorial shows how ISiCLE modifies MDL Molfiles to include theoretical isotropic shieldings and chemical shifts for each method. In our demonstration set, we added experimental details for each molecule including NMR spectra details, temperature, reference compounds, and solvents used. The user may modify the generated MDL Molfiles by including experimental data, as shown in tutorial. The section should begin with the string of “Experimental Shifts”, followed by the atom numbers, names, and their experimental shifts. The same atomic sequence must match the XYZ files. Note that the user should also enter the experimental values in the same format given in tutorial except experimental details; otherwise ISiCLE will skip it and eventually not read it.

# **S3. Demonstration Set**

We have compiled a molecule set of 312 compounds which spins a broad range of chemical types, classes, sizes and atom numbers from previous studies. The Table 1 shows the molecule sets used in this study, each set is named by the first author of the paper. It describes year of the paper, experimental conditions (frequency of ^1^H and ^13^C NMR Spectra, temperature), molecule types, number of molecules, nuclei, total and average number of ^1^H and ^13^C nuclei for each molecule set. Some molecules are shared by multiple authors.

**Table 1. Demonstration set sources and details.**

| Authors | Year | ^1^H NMR Spectra Frequency (MHz) | ^13^C NMR Spectra Frequency (MHz) | Temp (K) | # of molecules | Reported Nuclei | All Nuclei | Total # of atoms | Total # of ^1^H | Total # of ^13^C | Types of molecules |
| --- | --- | --- | --- | --- | --- | --- | --- | --- | --- | --- | --- |
| Alver et al. [[29](#_ENREF_29)] | 2011 | 500.13 | 125.76 | 300 | 1 | C, H | C, O, H, B | 24 | 11 | 8 | Boron-based compound |
| Asiri et al. [[30](#_ENREF_30)] | 2011 | 600 | 150 | 300 | 1 | C, H | C, H, O, N | 33 | 12 | 16 | Organic photochromic compounds |
| Bagno et al. [[31](#_ENREF_31)] | 2006 | N/A | N/A | 298 | 4 | C, H | C, H, O, N | 175 | 82 | 72 | Small organic molecules each having a single, well-defined conformation |
| Barkowski et al. [[32](#_ENREF_32)] | 2010 | N/A | N/A | 298 | 15 | C | C, H, O | 1243 | 756 | 459 | Pentacyclic terpenoids (fernenes) |
| Coruh et al. [[33](#_ENREF_33)] | 2010 | 500 | N/A | 298 | 1 | C, H | C, H, N, O, S | 25 | 8 | 13 | Heterocyclic aromatic compound and consists of flat five-membered ring |
| Fulmer et al. [[34](#_ENREF_34)] | 1997 | 300.1 | 75.5 | 298 | 33 | C, H | C, H, O, N, P, Cl, S | 464 | 279 | 137 | Commonly used NMR solvents |
| Hill et al. [[35](#_ENREF_35)] | 2014 | 400 | 100 | N/A | 1 | C, H | C, H, N, O, Cl | 70 | 33 | 30 | A complex drug molecule with multiple chemical groups and one stereocentre |
| Izgi et al. [[8](#_ENREF_8)] | 2007 | 500.13 | 125.76 | 300 | 1 | C, H | C, H, N | 24 | 15 | 8 | Molecule with cyclohexene (C_6_H_10_) attached to one of the carbon of ethylamine (C_2_H_7_N) |
| Karabacak et al. [[36](#_ENREF_36)] | 2009 | 300 | 75 | 298 | 1 | C, H | C, H, Cl, N | 17 | 8 | 7 | Molecule with three substituents such that B(OH)_2_ group and two F atoms (at meta- and ortho- positions) attached to the planar benzene ring. All forms are in the same plane. |
| Krishnakumar et al. [[37](#_ENREF_37)] | 2011 | N/A | N/A | 298 | 2 | C, H | C, H, N, O, Cl | 32 | 8 | 12 | An intermediate compound in the synthesis of agrochemicals with two planar rings |
| Krishnakumar et al. [[38](#_ENREF_38)] | 2012 | N/A | N/A | 298 | 2 | C, H | C, H, O, Cl | 32 | 14 | 14 | Nitrotoluene |
| Krishnakumar et al. [[39](#_ENREF_39)] | 2012 | N/A | N/A | 298 | 2 | C, H | C, H, O, N, Cl | 34 | 12 | 14 | Phenol derivatives |
| Kwan et al. [[10](#_ENREF_10)] | 2015 | 500 | N/A | 300 | 1 | C, H | C, H | 42 | 22 | 18 | Natural products |
| Li et al. [[40](#_ENREF_40)] | 2011 | N/A | N/A | N/A | 78 | C | C, H, N, Cl, O | 880 | 274 | 371 | A large amount of connection modes of substituents and groups. The amount of carbon in each molecule is not more than eight. |
| Lomas et al. [[41](#_ENREF_41)] | 2015 | N/A | N/A | 298 | 15 | H | Cl, H, O | 271 | 176 | 79 | Saturated alcohols |
| Osmialowski et al. [[42](#_ENREF_42)] | 2001 | N/A | N/A | 303 | 28 | C, H | C, H, N, O, Cl, F, Br | 787 | 318 | 383 | Molecules substituted 2-phenacylpyridines (ketimine forms) and their enolimine tautomers |
| Parlak et al. [[43](#_ENREF_43)] | 2008 | 500.13 | 125.76 | N/A | 1 | C, H | C, H, N, F | 26 | 4 | 12 | Polyfluoroaromatic compound with two rings |
| Perez et al. [[44](#_ENREF_44)] | 2006 | 400 | 300 | 300 | 2 | C, H | C, H, N, Cl | 44 | 20 | 16 | Chloropyrimidine species |
| Rablen et al. [[45](#_ENREF_45)] | 1999 | N/A | N/A | N/A | 80 | H | C, H, N, O, S, F, Si, Cl | 494 | 322 | 918 | Single conformer, rigid organic compounds |
| Sarotti et al. [[18](#_ENREF_18), [46](#_ENREF_46)] | 2009 & 2012 | 200.13 | N/A | N/A | 66 | C, H | C, H, N, O, Cl, S | 999 | 517 | 393 | Low polarity small-to-medium size compounds with a single conformer, and wide array of chemical functionalities and molecular complexity |
| Sebestian et al. [[47](#_ENREF_47)] | 2010 | 300 | 300 | 298 | 1 | C, H | C, H | 26 | 11 | 14 | Phenyl cyanide compound with two planar phenyl rings |
| Seca et al. [[22](#_ENREF_22)] | 2000 | 300.13 | 75.47 | N/A | 4 | C, H | C, H, O | 158 | 95 | 259 | Light petroleum extracts |
| Senyel et al. [[48](#_ENREF_48)] | 2008 | 500.13 | 125.76 | 300 | 1 | C, H | C, H, N | 23 | 13 | 9 | Structural element of many pharmaceutical drugs such as tolterodine and 3-phenoxy-3-phenylpropanamine |
| Senyel et al. [[49](#_ENREF_49)] | 2008 | 500.13 | 125.76 | 300 | 1 | C, H | C, H, N | 28 | 18 | 8 | 3-Piperidino-propylamine molecule consists of piperidine (C_5_H_11_N) attached to one of the carbon of propylamine (C_3_H_9_N) |
| Sridevi et al. [[50](#_ENREF_50)] | 2011 | 500 | 125 | N/A | 1 | C, H | C, H, N, O | 213 | 8 | 10 | Chromene, a planar compound with two rings |
| Tormena et al. [[51](#_ENREF_51)] | 2004 | 500.13 | 125.78 | 300 | 3 | C, H | C, H, N, O | 52 | 25 | 25 | Para-X-substituted (X = H, CH3O and NO2) aromatic carbonyl compounds |
| Vijaja et al. [[52](#_ENREF_52)] | 2015 | 500 | 500 | 300 | 1 | C, H | C, H, N, O | 47 | 24 | 20 | A novel unsymmetrical azine which has acceptor and donor groups in the para positions of the benzene rings on opposing ends of the hyperpolarizable electron system |
| Watts et al. [[23](#_ENREF_23)] | 2011 | N/A | N/A | 298 | 6 | C, H | C, H, O | 271 | 126 | 110 | Coniferyl alcohol, a monomeric building block of lignin, different stereoisomers and conformers |
| Wiitala et al. [[53](#_ENREF_53)] | 2006 | varying | varying | N/A | 43 | C, H | C, H, O, S, Br, N, Cl | 484 | 269 | 141 | Organic compounds |
| Wiitala et al. [[54](#_ENREF_54)] | 2006 | 500 | 500 | 300 | 7 | C, H | C, H, N, O, S | 170 | 98 | 58 | Cis and trans forms of 2-, 3-, and 4-methylcyclohexanols |
| Willoughby et al. [[55](#_ENREF_55)] | 2004 | 500 | 500 | N/A | 2 | C, H | C, H, O | 44 | 28 | 14 | Cis- and trans-diastereomers of 3-methylcyclohexanol |
| Yang et al. [[21](#_ENREF_21)] | 2008 | N/A | N/A | N/A | 2 | C | C, H, O | 114 | 56 | 46 | Complex natural products |

The full list of demonstration set is found in the supplemental spreadsheet, named DemonstrationSet.xlsx. In that file, the molecules are given with InChI, InChIKeys and SMILES. Molecular formula, molecular weight, and chemical classes obtained with ClassyFire [[56](#_ENREF_56)] are also given.

XYZ, 2D and 3D MDL MOL files, and NWChem output files for each run can be also found in S1.

# **S4. Error analysis of chemical shift calculations for the demonstration set**

A total of 2,494 carbon nuclei and 3,127 hydrogen nuclei were calculated for all 312 molecules of the demonstration set and compared with experimental data. Deviation bars indicating mean absolute error (MAE) and maximum absolute error (MAXAE) are plotted for each method in Figure 1.

For ^13^C, the MAE of each method with cc-pVTZ (7-10 ppm) is higher than those with cc-pVDZ (5-6 ppm). MAE of methods with a larger basis set deviate more compared to those with a smaller basis set. The smallest deviations are observed for B3LYP and B35LYP, both in MAE and MAXAE results. The same situation is observed for ^1^H NMR chemical shifts as well: MAE of each method with cc-pVTZ (~0.35 ppm) is higher than those with cc-pVDZ (~ 0.30 ppm). In contrast to ^13^C NMR chemical shifts, ^1^H NMR chemical shifts are better predicted with methods using larger basis sets (cc-pVTZ). Although the error differences among each method may be too low to confidently identify the outperforming method, B3LYP/cc-pVDZ is the most successful combination in the calculation of ^1^H NMR chemical shifts for our application shown here.


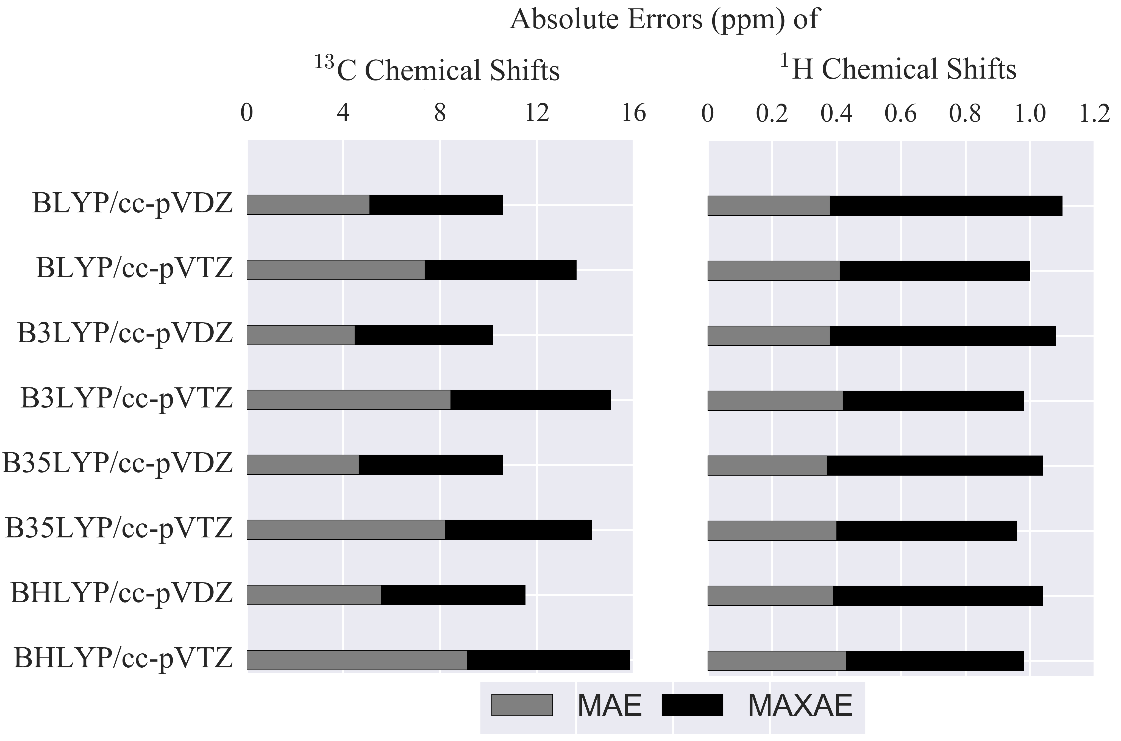


**Figure 1. Mean absolute errors (MAE) and maximum absolute errors (MAXAE) of chemical shifts for the demonstration set.** The grey bars represent MAE, the black bars represent MAXAE. For all methods, geometries are optimized at B3LYP/6-31G(d) in chloroform.

# **S5. Performance vs. computational cost**

We found that the smaller basis set (cc-pVDZ) used in the calculation of both ^13^C and ^1^H NMR chemical shifts was an excellent compromise between accuracy and computational performance, compared to the larger cc-pVTZ basis. This finding is similar to a recent benchmark study [[57](#_ENREF_57)] that showed B3LYP/cc-pVDZ is a reliable combination, balancing accuracy with computational cost in ^13^C chemical shifts calculation. Figure 2 shows the computational cost of each method. The larger basis set (cc-pVTZ) took 2-3 times longer to complete than cc-pVDZ (in terms of total CPU time).


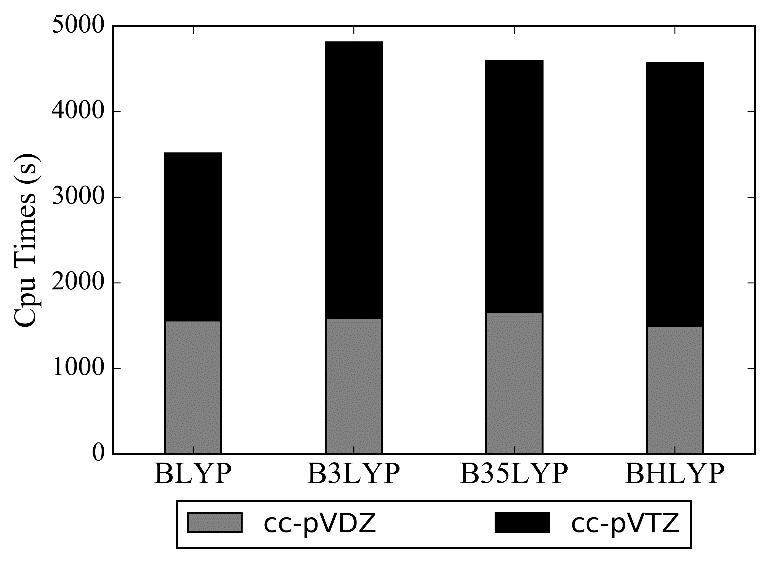


**Figure 2. Computational costs of DFT methods performed for the demonstration set.** Each bar is for two DFT methods with basis sets of cc-pVDZ and cc-pVTZ. The grey bars represent CPU times for the methods with cc-pVDZ and the black bars represent those with cc-pVTZ.

**S6. Application of Empirical Scaling Derived from Linear Regression Approach**

The linear regression parameters were determined for each level of theory mentioned in the paper. In Figures 3-10, the linear correlation plots are given for experimental chemical shifts vs. computed chemical shifts and experimental chemical shifts vs. computed isotropic shielding constants for both ^13^C and ^1^H chemical shift data. The chemical shifts are scaled using the linear equations given on each regarding plot.

**Table 2. Average results of unscaled and scaled ^13^C chemical shifts for each DFT method.**

|  | Unscaled | | | | Scaled | | | |
| --- | --- | --- | --- | --- | --- | --- | --- | --- |
| Method* | MAE | ME | RMSE | MAXAE | MAE | ME | RMSE | MAXAE |
| I | 5.29 | 2.26 | 7.22 | 54.94 | 4.68 | 0.00 | 6.53 | 56.16 |
| II | 7.37 | 6.71 | 8.56 | 50.47 | 4.17 | 0.00 | 5.62 | 57.60 |
| III | 4.57 | 3.39 | 6.54 | 39.69 | 3.88 | 0.04 | 5.62 | 36.63 |
| IV | 8.43 | 7.45 | 9.95 | 46.35 | 3.86 | 0.00 | 5.99 | 41.14 |
| V | 4.89 | 3.49 | 7.66 | 50.41 | 4.04 | 0.00 | 6.79 | 54.21 |
| VI | 7.38 | -6.71 | 9.04 | 51.19 | 4.17 | 0.00 | 5.93 | 57.60 |
| VII | 5.77 | 4.48 | 8.13 | 48.45 | 3.86 | 0.00 | 6.52 | 53.96 |
| VIII | 8.98 | 8.07 | 10.77 | 50.62 | 3.48 | 0.00 | 5.61 | 55.83 |

* I - BLYP/CC-PVDZ//B3LYP/6-31G(d)/CH/CHCL3, II - BLYP/CC-PVTZ//B3LYP/6-31G(d)/CH/CHCL3, III - B3LYP/CC-PVDZ//B3LYP/6-31G(d)/CH/CHCL3, IV - B3LYP/CC-PVTZ//B3LYP/6-31G(d)/CH/CHCL3, V - B35LYP/CC-PVDZ//B3LYP/6-31G(d)/CH/CHCL3, VI - B35LYP/CC-PVTZ//B3LYP/6-31G(d)/CH/CHCL3, VII - BHLYP/CC-PVDZ//B3LYP/6-31G(d)/CH/CHCL3, VIII - BHLYP/CC-PVTZ//B3LYP/6-31G(d)/CH/CHCL3.

**Table 3. Average results of unscaled and scaled ^1^H chemical shifts for each DFT method.**

|  | Unscaled | | | | Scaled | | | |
| --- | --- | --- | --- | --- | --- | --- | --- | --- |
| Method* | MAE | ME | RMSE | MAXAE | MAE | ME | RMSE | MAXAE |
| I | 0.33 | 0.07 | 0.65 | 9.99 | 0.33 | 0.00 | 0.65 | 9.97 |
| II | 0.36 | 0.13 | 0.62 | 9.08 | 0.30 | 0.00 | 0.59 | 9.39 |
| III | 0.32 | 0.06 | 0.58 | 10.00 | 0.31 | 0.00 | 0.62 | 10.03 |
| IV | 0.35 | 0.11 | 0.61 | 9.09 | 0.29 | 0.00 | 0.57 | 9.42 |
| V | 0.32 | 0.08 | 0.63 | 10.06 | 0.30 | 0.00 | 0.64 | 10.18 |
| VI | 0.32 | -0.08 | 0.63 | 10.06 | 0.56 | 0.00 | 0.79 | 10.58 |
| VII | 0.33 | 0.07 | 0.63 | 9.99 | 0.30 | 0.00 | 0.60 | 10.06 |
| VIII | 0.36 | 0.10 | 0.61 | 9.07 | 0.28 | 0.00 | 0.56 | 9.43 |

* I - BLYP/CC-PVDZ//B3LYP/6-31G(d)/CH/CHCL3, II - BLYP/CC-PVTZ//B3LYP/6-31G(d)/CH/CHCL3, III - B3LYP/CC-PVDZ//B3LYP/6-31G(d)/CH/CHCL3, IV - B3LYP/CC-PVTZ//B3LYP/6-31G(d)/CH/CHCL3, V - B35LYP/CC-PVDZ//B3LYP/6-31G(d)/CH/CHCL3, VI - B35LYP/CC-PVTZ//B3LYP/6-31G(d)/CH/CHCL3, VII - BHLYP/CC-PVDZ//B3LYP/6-31G(d)/CH/CHCL3, VIII - BHLYP/CC-PVTZ//B3LYP/6-31G(d)/CH/CHCL3.

The mean absolute error (MAE), mean unsigned error (ME), root-mean-square-error (RMSE) and maximum absolute error (MAXAE) results for both scaled and unscaled ^13^C and ^1^H chemical shifts are reported for each DFT method in Table 2 and Table 3, respectively. Note that outliers are discarded.

The reported slope, intercept and correlation coefficient values of individual molecule sets can be found in the MoleculeSets_LinearRegressionParameters.xlsx file.

**Figure 3. Linear correlation plots of ^13^C and ^1^H NMR chemical shifts calculated using the DFT method: GIAO/BLYP/cc-pVDZ//B3LYP/6-31G(d) for the demonstration set in CHCl_3_.**

**Figure 4. Linear correlation plots of ^13^C and ^1^H NMR chemical shifts calculated using the DFT method: GIAO/BLYP/cc-pVTZ//B3LYP/6-31G(d) for the demonstration set in CHCl_3_.**

**Figure 5. Linear correlation plots of ^13^C and ^1^H NMR chemical shifts calculated using the DFT method: GIAO/B3LYP/cc-pVDZ//B3LYP/6-31G(d) for the demonstration set in CHCl_3_.**

**Figure 6. Linear correlation plots of ^13^C and ^1^H NMR chemical shifts calculated using the DFT method: GIAO/B3LYP/cc-pVTZ//B3LYP/6-31G(d) for the demonstration set in CHCl_3_.**

**Figure 7. Linear correlation plots of ^13^C and ^1^H NMR chemical shifts calculated using the DFT method: GIAO/B35LYP/cc-pVDZ//B3LYP/6-31G(d) for the demonstration set in CHCl_3_.**

**Figure 8. Linear correlation plots of ^13^C and ^1^H NMR chemical shifts calculated using the DFT method: GIAO/B35LYP/cc-pVTZ//B3LYP/6-31G(d) for the demonstration set inCHCl_3_.**

**Figure 9. Linear correlation plots of ^13^C and ^1^H NMR chemical shifts calculated using the DFT method: GIAO/BHLYP/cc-pVDZ//B3LYP/6-31G(d) for the demonstration set in CHCl_3_.**

**Figure 10. Linear correlation plots of ^13^C and ^1^H NMR chemical shifts calculated using the DFT method: GIAO/BHLYP/cc-pVTZ//B3LYP/6-31G(d) for the demonstration set in CHCl_3_.**

# **S7. Application of Empirical Scaling to Functional Groups**

**Table 4. Linear regression parameters for subgroups of carbon and hydrogen nuclei.**

| Groups | Mean | Median | Max | Number of atoms | Slope | Intercept | R^2^ |
| --- | --- | --- | --- | --- | --- | --- | --- |
| C-O | 3.15 | 2.16 | 32.51 | 251 | 0.99 | 2.70 | 0.99 |
| C-Cl | 11.23 | 10.59 | 39.69 | 149 | 0.95 | 16.8926 | 0.82 |
| C-F | 2.79 | 2.79 | 3.14 | 8 | 1.08 | -7.95 | 0.52 |
| C-N | 4.23 | 2.61 | 23.61 | 199 | 0.97 | 7.07 | 0.98 |
| C-S | 5.77 | 4.71 | 16.54 | 20 | 0.98 | 7.36 | 0.99 |
| C-H | 3.88 | 2.79 | 24.95 | 1161 | 0.97 | 5.18 | 0.99 |
| C-C | 4.66 | 3.19 | 51.64 | 1709 | 0.98 | 5.60 | 0.98 |
| H-C | 0.28 | 0.17 | 4.41 | 1793 | 1.01 | -0.07 | 0.96 |
| H-O | 2.27 | 1.81 | 10.00 | 41 | 0.80 | -0.66 | 0.51 |
| H-N | 0.72 | 0.51 | 2.26 | 17 | 1.03 | -0.80 | 0.78 |
| H-S | 0.13 | 0.13 | 0.13 | 1 |  |  |  |

**Table 5. Linear regression parameters for carbon nuclei with excluded nucleus.**

|  | Number of atoms | Slope | Intercept | R^2^ |
| --- | --- | --- | --- | --- |
| w/out O | 1393 | 0.97 | 6.52 | 0.96 |
| w/out Cl | 1485 | 0.95 | 6.82 | 0.96 |
| w/out F | 1617 | 0.96 | 6.82 | 0.95 |
| w/out N | 1439 | 0.96 | 6.76 | 0.95 |
| w/out S | 1608 | 0.96 | 6.79 | 0.95 |
| w/out H | 527 | 0.91 | 16.88 | 0.90 |
| w/out C | 1393 | 0.97 | 6.52 | 0.96 |

**Table 6. Linear regression parameters for hydrogen nuclei with excluded nucleus.**

|  | Number of atoms | Slope | Intercept | R^2^ |
| --- | --- | --- | --- | --- |
| w/out C | 59 | 0.78 | -0.44 | 0.57 |
| w/out O | 1709 | 1.00 | -0.04 | 0.93 |
| w/out N | 1730 | 0.96 | 0.05 | 0.90 |

Linear regression parameters applied to different groups of ^13^C and ^1^H chemical shifts (calculated using the outperforming DFT method: GIAO/B3LYP/cc-pVDZ//B3LYP/6-31G(d) in CHCl_3_) are reported in Figure 4, with the mean, median and maximum values given in Figure 7. Moreover, in order to determine the effect of an individual nucleus (oxygen (O), chlorine (Cl), fluorine (F), hydrogen (H), nitrogen (N), oxygen (O), or sulfur (S)) on the accuracy of the ^13^C chemical shifts, the scaling procedure was applied to the molecules without having the specified nuclei if bonded to carbon. The same procedure was applied to the molecules in which carbon (C), oxygen, or nitrogen nuclei were excluded if attached to hydrogen. Slope, intercept, and correlation coefficient values are listed in Figure 5 and Figure 6 for carbon and hydrogen nuclei, respectively.

# **S8. Tetramethylsilane**

Tetramethylsilane (TMS) is the most common reference compound for ^13^C and ^1^H nuclei and constitutes a good standard for experimental NMR spectroscopy [[46](#_ENREF_46)]. IUPAC has published a few recommendations regarding the reporting of NMR chemical shifts, including TMS serving as a universal reference for reporting ^1^H and ^13^C NMR chemical shifts [[58](#_ENREF_58)]. A recent publication [[59](#_ENREF_59)] discusses the effect of temperature, pressure, and solvent change on TMS and lists the IUPAC recommendations for referencing and reporting NMR chemical shifts in solids for best practice. Although the use of multi-referencing (i.e. benzene – methanol) has been found better for accuracy of ^13^C NMR chemical shifts, [[60-62](#_ENREF_60)], we have preferred TMS for reporting NMR chemical shifts for our validation set since the experimental ^13^C and ^1^H NMR chemical shifts of TMS are zero [[63](#_ENREF_63)]. Also, TMS is a simple and easy-to-model molecule whose NMR chemical shifts are well known in chloroform. The magnitude of change in isotopic shieldings in TMS changes with solvent, but change of the proton chemical shift in TMS with chloroform is zero at room temperature under standard pressure [[64](#_ENREF_64)]. Other commonly used solvents (e.g. methanol, dmso, benzene) affect proton chemical shift in TMS [[65](#_ENREF_65)].

In Table 7, slope, intercept, and correlation coefficient values for the plots of experimental shifts versus calculated shielding tensors are reported for carbon and hydrogen nuclei. When compared TMS shielding tensors with intercept values, the absolute deviations are 1.67 to 15.08 ppm and 0.62 to 2.68 ppm for carbon and hydrogen nuclei, respectively.

**Table 7. Linear regression parameters for the plots of experimental shifts vs. shielding tensors for carbon and hydrogen.**

|  | Carbon | | | | Hydrogen | | | |
| --- | --- | --- | --- | --- | --- | --- | --- | --- |
| Method* | Slope | Intercept | R^2^ | TMS | Slope | Intercept | R^2^ | TMS |
| I | -1.04 | 192.24 | 0.98 | 190.57 | -0.98 | 30.85 | 0.93 | 31.47 |
| II | -0.98 | 169.33 | 0.99 | 177.65 | -0.94 | 29.64 | 0.94 | 31.55 |
| III | -1.01 | 191.38 | 0.99 | 194.00 | -0.95 | 30.30 | 0.93 | 31.52 |
| IV | -0.95 | 169.75 | 0.98 | 181.54 | -0.92 | 29.31 | 0.94 | 31.57 |
| V | -0.99 | 190.42 | 0.98 | 195.32 | -0.95 | 30.35 | 0.94 | 31.67 |
| VI | -0.94 | 171.50 | 0.99 | 183.40 | -0.92 | 29.37 | 0.95 | 31.71 |
| VII | -0.96 | 189.82 | 0.98 | 198.18 | -0.93 | 29.84 | 0.94 | 31.61 |
| VIII | -0.91 | 171.37 | 0.97 | 186.45 | -0.91 | 28.94 | 0.95 | 31.62 |

* I - BLYP/CC-PVDZ//B3LYP/6-31G(d)/CH/CHCL3, II - BLYP/CC-PVTZ//B3LYP/6-31G(d)/CH/CHCL3, III - B3LYP/CC-PVDZ//B3LYP/6-31G(d)/CH/CHCL3, IV - B3LYP/CC-PVTZ//B3LYP/6-31G(d)/CH/CHCL3, V - B35LYP/CC-PVDZ//B3LYP/6-31G(d)/CH/CHCL3, VI - B35LYP/CC-PVTZ//B3LYP/6-31G(d)/CH/CHCL3, VII - BHLYP/CC-PVDZ//B3LYP/6-31G(d)/CH/CHCL3, VIII - BHLYP/CC-PVTZ//B3LYP/6-31G(d)/CH/CHCL3.

# **S9. Monte Carlo Cross-validation**

As a final assessment for the data collected with the demonstration set, we assessed the stability and accuracy of the linear regression approach using cross-validation [[66](#_ENREF_66)]. Cross-validation is a technique mostly used in prediction problems to evaluate how well a model trained on a subset of the data generalizes to another, independent subset. Specifically, we performed Monte Carlo cross-validation [[67](#_ENREF_67), [68](#_ENREF_68)]. We randomly split our demonstration set into 50% training set, 50% validation set. Next, we fit a linear model to the training set, assessing the accuracy of this model to predict ^13^C and ^1^H NMR chemical shifts. We then evaluated the performance of the predictive model by estimating the model parameters, slope and intercept, over the iterations of 10,000 times (to account for noise and make a fair assessment) (***Figure 11***). The deviations of each slope and intercept values show us how independent the predicted linear model is in overall for our demonstration set.

Slope values ranged from 0.97 to 0.99 for ^13^C NMR chemical shifts and from 0.99 to 1.03 for ^1^H NMR chemical shifts, which are quite close to unity. When we fit the parameters to all ^13^C NMR chemical shifts (i.e. without splitting into two sets), the correlation coefficient improved, with a decrease in average standard deviation from 0.0018 to 0.0004 ppm. In overall, the little variations between slopes and intercepts show that linear regression parameters are stable and sufficiently fair to properly estimate model prediction performance of ^13^C and ^1^H NMR chemical shifts for the demonstration set. Compared to carbons, proton chemical shifts have a larger deviation in slope values showing a smaller deviation in intercept values. The scaled ^1^H NMR chemical shifts disperse from the mean, which explains the increasing trend in cumulative standard deviation.

To the best of our knowledge, we employed this analysis on the largest set of carbon chemical shifts available to-date, increasing our confidence to report the cross-validated slope and intercept ranges.


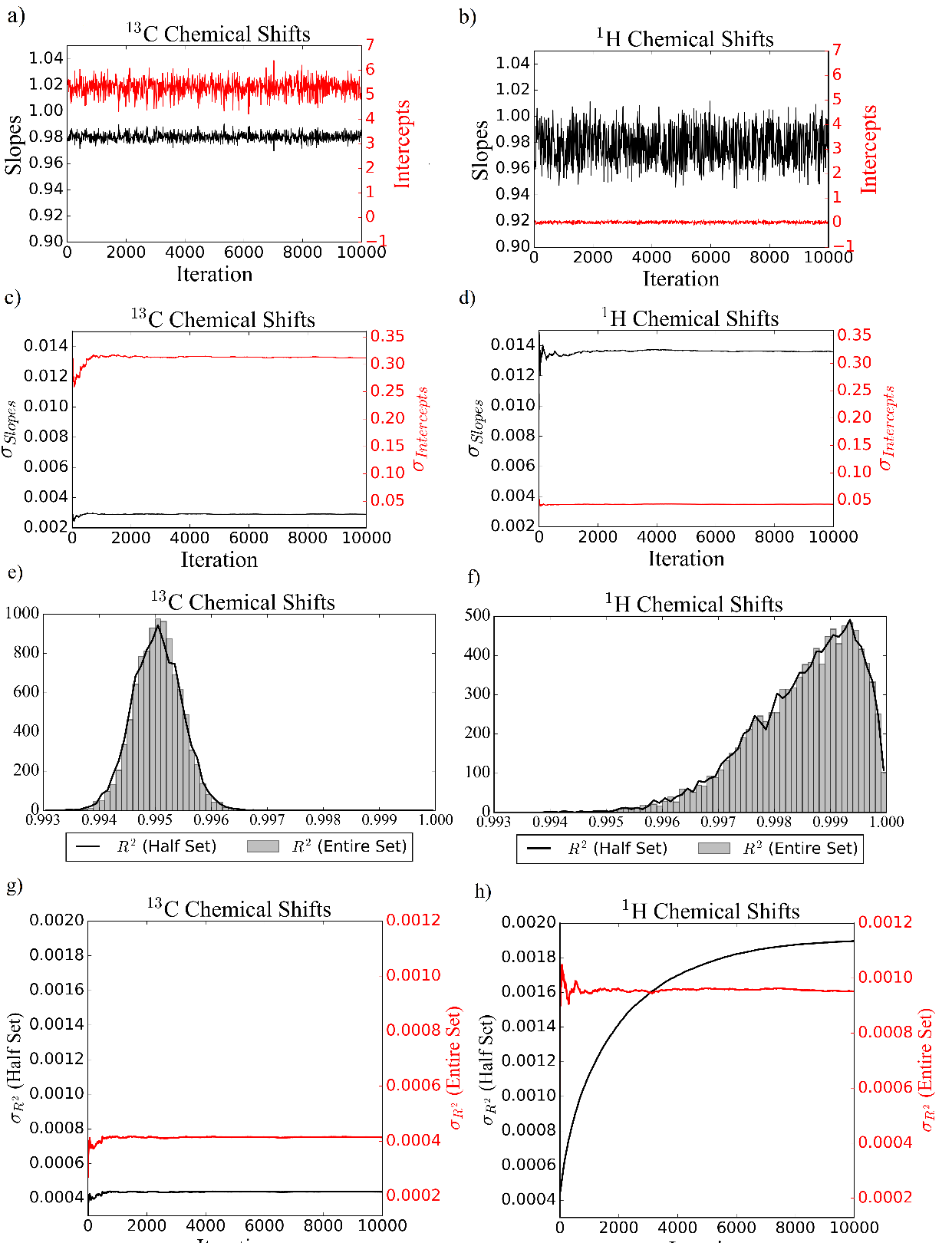


**Figure 11. Monte Carlo cross-validation results**. a, c, e, g) ^13^C NMR chemical shifts. b, d, f, h) ^1^H NMR chemical shifts. a, b) Slope and intercept profiles. c, d) Cumulative standard deviation profiles of slope and intercept values. e, f) Correlation coefficients of all linear models. g, h) Cumulative standard deviation profiles of correlation coefficients.

# **S10. Conformational Analysis of Methylcyclohexane**

The initial Cartesian coordinates of axial and equatorial methylcyclohexane are given in Table 2 and Table 3. The geometries are taken from the study of Willoughby et al. [[55](#_ENREF_55)]. The XYZ and 3D MOL files of 80 conformers (40 axial & 40 equatorial) can be found in the folder of S1.

**Table 8. Initial geometry of axial methylcyclohexane [**[**55**](#_ENREF_55)**]**

| Atom | X - coordinate | Y - coordinate | Z - coordinate |
| --- | --- | --- | --- |
| C | 1.124582 | 0.000000 | 0.496581 |
| C | 0.252416 | 1.259218 | 0.63716 |
| C | -0.937284 | 1.262901 | -0.329215 |
| C | -1.786563 | 0.000000 | -0.157728 |
| C | -0.937284 | -1.262901 | -0.329215 |
| C | 0.252415 | -1.259217 | 0.637161 |
| C | 1.926795 | 0.000000 | -0.808932 |
| H | 1.848685 | 0.000000 | 1.322358 |
| H | -0.134168 | 1.306571 | 1.664983 |
| H | 0.86723 | 2.155583 | 0.488804 |
| H | -1.548219 | 2.156977 | -0.161744 |
| H | -0.578781 | 1.32098 | -1.365572 |
| H | -2.227121 | -0.000001 | 0.849875 |
| H | -2.620311 | 0.000000 | -0.868718 |
| H | -1.548219 | -2.156977 | -0.161746 |
| H | -0.578779 | -1.320979 | -1.365572 |
| H | -0.13417 | -1.306569 | 1.664983 |
| H | 0.867229 | -2.155583 | 0.488806 |
| H | 2.567922 | -0.885698 | -0.869795 |
| H | 1.280315 | 0.000004 | -1.691737 |
| H | 2.567927 | 0.885694 | -0.869791 |

**Table 9. Initial geometry of equatorial methylcyclohexane [**[**55**](#_ENREF_55)**]**

| Atom | X - coordinate | Y - coordinate | Z - coordinate |
| --- | --- | --- | --- |
| C | -1.012353 | 0.000000 | -0.334045 |
| C | -0.292983 | 1.256686 | 0.169311 |
| C | 1.189311 | 1.261608 | -0.213784 |
| C | 1.893649 | 0.000000 | 0.2923 |
| C | 1.189311 | -1.261609 | -0.213784 |
| C | -0.292983 | -1.256686 | 0.169311 |
| C | -2.483368 | -0.000001 | 0.074989 |
| H | -0.956168 | 0.000000 | -1.434615 |
| H | -0.79219 | 2.152239 | -0.220945 |
| H | -0.384179 | 1.295857 | 1.26597 |
| H | 1.277566 | 1.307647 | -1.30857 |
| H | 1.680325 | 2.157835 | 0.181435 |
| H | 2.945044 | 0.000001 | -0.016108 |
| H | 1.883908 | 0.000000 | 1.391659 |
| H | 1.277568 | -1.307649 | -1.30857 |
| H | 1.680325 | -2.157835 | 0.181437 |
| H | -0.79219 | -2.152238 | -0.220945 |
| H | -0.384178 | -1.295856 | 1.26597 |
| H | -3.001779 | -0.886391 | -0.305134 |
| H | -2.575775 | 0.000001 | 1.167763 |
| H | -3.00178 | 0.886389 | -0.305135 |

Energies (thermal correction to enthalpy, total entropy, total DFT energy, Gibb’s free energy, Relative Gibb’s free energy, and Boltzmann distribution factors) are reported for each conformer in the file Conformational_analysis_of_methylcyclohexane.xlsx in S1. Calculations of Boltzmann-weighted ^13^C and ^1^H NMR chemical shifts are given in the same file.

**Table 10. NMR shielding tensors of axial and equatorial methylcyclohexane**

|  |  | Axial | | Equatorial | |
| --- | --- | --- | --- | --- | --- |
| Atom # | Atom Type | Willoughby et al. [[55](#_ENREF_55)] | NWChem | Willoughby et al. [[55](#_ENREF_55)] | NWChem |
| 1 | C | 149.248 | 149.0061 | 144.2576 | 144.1113 |
| 2 | C | 146.8882 | 146.7424 | 143.2005 | 143.1215 |
| 3 | C | 158.2499 | 158.1207 | 151.9004 | 151.8021 |
| 4 | C | 151.3094 | 151.1607 | 152.2265 | 152.1083 |
| 5 | C | 158.2499 | 158.1207 | 151.9004 | 151.8011 |
| 6 | C | 146.8881 | 146.7423 | 143.2004 | 143.1213 |
| 7 | C | 164.8813 | 164.7805 | 158.6446 | 158.6293 |
| 8 | H | 29.9669 | 29.9491 | 30.5637 | 30.54 |
| 9 | H | 30.2923 | 30.2822 | 30.1884 | 30.1799 |
| 10 | H | 30.3477 | 30.3524 | 30.9973 | 30.9824 |
| 11 | H | 30.4845 | 30.4919 | 30.5862 | 30.572 |
| 12 | H | 30.2912 | 30.2687 | 30.1443 | 30.1471 |
| 13 | H | 30.632 | 30.6316 | 30.1939 | 30.1796 |
| 14 | H | 30.1776 | 30.1788 | 30.7141 | 30.7034 |
| 15 | H | 30.4845 | 30.4919 | 30.5862 | 30.5719 |
| 16 | H | 30.2912 | 30.2687 | 30.1443 | 30.1472 |
| 17 | H | 30.2923 | 30.2822 | 30.1884 | 30.1799 |
| 18 | H | 30.3477 | 30.3524 | 30.9973 | 30.9825 |
| 19 | H | 31.1057 | 31.1189 | 30.8821 | 30.891 |
| 20 | H | 30.3142 | 30.2824 | 31.289 | 31.2838 |
| 21 | H | 31.1057 | 31.1189 | 30.882 | 30.8909 |

NMR isotropic shielding tensors both used in our study and taken from the study of Willoughby et al. are reported in Table 10 for all carbon and hydrogen atoms for axial and equatorial methylcyclohexane.

**Table 11. ^1^H computed NMR chemical shifts of axial and equatorial methylcyclohexane**

|  | Axial | | Equatorial | |
| --- | --- | --- | --- | --- |
| Atoms | Computed ^1^H chemical shifts [[55](#_ENREF_55)] | Computed ^1^H chemical shifts | Computed ^1^H chemical shifts [[55](#_ENREF_55)] | Computed ^1^H chemical shifts |
| H1 | 1.84 | 1.9255 | 1.29 | 1.3346 |
| H2b | 1.54 | 1.5924 | 1.63 | 1.6947 |
| H2a | 1.49 | 1.5222 | 0.88 | 0.8922 |
| H3b | 1.36 | 1.3827 | 1.26 | 1.3026 |
| H3a | 1.54 | 1.6059 | 1.67 | 1.7275 |
| H4b | 1.22 | 1.243 | 1.63 | 1.695 |
| H4a | 1.64 | 1.6958 | 1.15 | 1.1712 |
| Me1 | 1.03 | 1.0345 | 0.86 | 0.8527 |
| Me2 | 1.03 | 1.0345 | 0.86 | 0.8527 |
| Me3 | 1.03 | 1.0345 | 0.86 | 0.8527 |

The shieldings are calculated at B3LYP/6-311+G(2d,p)//M06-2X/6-31+G(d,p) and referenced to TMS. Computed ^1^H chemical shifts both used in our study and taken from the study of Willoughby et al. are reported in Table 11 for axial and equatorial methylcyclohexane.

**Table 12. Experimental and Boltzmann-weighted and scaled ^1^H chemical shifts (ppm) of methylcyclohexane calculated at B3LYP/6-311+G(2d,p)//M06-2X/6-31+G(d,p).** I: Absolute deviation (ppm) in Boltzmann weighted ^1^H chemical shifts. II: Absolute deviation (ppm) reported [[55](#_ENREF_55)]. III: Absolute deviation (ppm) in scaled ^1^H chemical shifts.

| Atoms | Experimental ^1^H chemical shifts [[55](#_ENREF_55)] | Boltzmann-weighted ^1^H chemical shifts [[55](#_ENREF_55)] | Boltzmann-weighted ^1^H chemical shifts | I [[55](#_ENREF_55)] | II | III |
| --- | --- | --- | --- | --- | --- | --- |
| H1 | 1.34 | 1.3 | 1.342355045 | 0.01 | 0.04 | 0.02 |
| H2b | 1.65 | 1.63 | 1.693357402 | 0.04 | 0.02 | 0.01 |
| H2a | 0.88 | 0.9 | 0.900468198 | 0.03 | 0.02 | 0.01 |
| H3b | 1.23 | 1.27 | 1.303651242 | 0.08 | 0.04 | 0.04 |
| H3a | 1.68 | 1.67 | 1.725904107 | 0.04 | 0.01 | 0.01 |
| H4b | 1.62 | 1.62 | 1.689067896 | 0.06 | 0.00 | 0.00 |
| H4a | 1.14 | 1.16 | 1.178084915 | 0.05 | 0.02 | 0.02 |
| Me1 | 0.86 | 0.87 | 0.855085966 | 0.00 | 0.01 | 0.01 |
| Me2 | 0.86 | 0.87 | 0.855085966 | 0.00 | 0.01 | 0.01 |
| Me3 | 0.86 | 0.87 | 0.855085966 | 0.00 | 0.01 | 0.01 |
|  |  |  | MAE = | 0.031 | 0.018 | 0.017 |

Experimental and Boltzmann-weighted ^1^H chemical shifts used in our study and taken from Willoughby et al. are reported in Table 12 for methylcyclohexane. The Boltzmann-weighted ^1^H chemical shifts are compared with experimental chemical shifts. The MAE is 0.032 ppm for ^1^H chemical shifts while it is 0.018 ppm in Willoughby et al. It decreases to 0.017 ppm when the Boltzmann-weighted chemical shifts are scaled using the following equation$\delta_{exp}=0.9501\times\delta_{comp}+0.0307$ obtained from Figure 12.

**Figure 12. Linear correlation plot of experimental and Boltzmann-weighted ^1^H NMR chemical shifts (ppm) calculated at GIAO/ B3LYP/6-311+G(2d,p)//M06-2X/6-31+G(d,p) level of theory.**

**Table 13. Experimental and calculated at B3LYP/6-311+G(2d,p)//M06-2X/6-31+G(d,p) (ppm) of methylcyclohexane.**

| Atoms | Experimental ^13^C chemical shifts [[69](#_ENREF_69)] | Experimental ^13^C chemical shifts [[70](#_ENREF_70)] | Computed ^13^C chemical shifts | |
| --- | --- | --- | --- | --- |
|  |  |  | Axial | Equatorial |
| C-Me | 23.1 | 22.95 | 18.9112 | 25.0518 |
| C1 | 33.1 | 32.91 | 34.5804 | 39.4893 |
| C2 | 35.8 | 35.62 | 36.886 | 40.4997 |
| C3 | 26.8 | 26.6 | 25.5167 | 31.8236 |
| C4 | 26.7 | 26.5 | 32.4857 | 31.5106 |

Experimental ^13^C chemical shifts reported from [[67](#_ENREF_67), [68](#_ENREF_68)] and calculated ^13^C chemical shifts for axial and equatorial methylcyclohexane are given in Table 13.

**Table 14. Boltzmann-weighted and scaled ^13^C chemical shifts (ppm) of methylcyclohexane. I: Absolute deviation (ppm) in Boltzmann weighted ^13^C chemical shifts. II: Absolute deviation (ppm) in scaled ^13^C chemical shifts.**

| Atoms | Boltzmann-weighted ^13^C chemical shifts | Scaled ^13^C chemical shifts ^a^ | Scaled ^13^C chemical shifts ^b^ | I ^c^ | I ^d^ | II ^e^ | II ^f^ |
| --- | --- | --- | --- | --- | --- | --- | --- |
| C-Me | 24.8 | 22.1 | 22.0 | 1.7 | 1.9 | 0.9 | 1.0 |
| C1 | 39.3 | 33.7 | 33.5 | 6.2 | 6.4 | 0.6 | 0.6 |
| C2 | 40.3 | 34.5 | 34.4 | 4.5 | 4.8 | 1.2 | 1.3 |
| C3 | 31.6 | 27.5 | 27.4 | 4.8 | 5.0 | 0.7 | 0.8 |
| C4 | 31.5 | 27.5 | 27.3 | 4.8 | 5.0 | 0.8 | 0.8 |
|  |  |  | MAE = | 4.4 | 4.6 | 0.8 | 0.9 |

^a^ Scaled ^13^C chemical shifts using the ref [[69](#_ENREF_69)] with the equation $\delta_{exp}=0.79\times\delta_{comp}+2.22$

^b^ Scaled ^13^C chemical shifts using the ref [[70](#_ENREF_70)] with the equation $\delta_{exp}=0.79\times\delta_{comp}+2.36$

^c-d^ Absolute deviation (ppm) in Boltzmann weighted ^13^C chemical shifts when compared to experimental chemical shifts taken from the ref [[69](#_ENREF_69)]^c^ and [[70](#_ENREF_70)]^d^

^e-f^ Absolute deviation (ppm) in scaled ^13^C chemical shifts obtained using the ref [[69](#_ENREF_69)]^e^ and [[70](#_ENREF_70)]^f^

In Table 14, Boltzmann-weighted and scaled ^13^C chemical shifts are compared with the two experimental data. The MAE is 4.4 and 4.6 ppm and decreases to 0.9 and 0.8 ppm when the chemical shifts are scaled with the following equations $\delta_{exp}=0.79\times\delta_{comp}+2.22$ and $\delta_{exp}=0.79\times\delta_{comp}+2.36$, respectively, obtained from Figure 13.

**Figure 13. Linear correlation plot of experimental (taken from Ref [70] (black) and Ref [69] (red)) and Boltzmann-weighted ^13^C NMR chemical shifts (ppm) calculated at GIAO/ B3LYP/6-311+G(2d,p)//M06-2X/6-31+G(d,p) level of theory.**

**References**

1. *Fabric*. June 5 2018]; Available from: <http://www.fabfile.org/> and <https://github.com/fabric/fabric/>.

2. Williams, A.J., *A perspective of publicly accessible/open-access chemistry databases.* Drug Discov Today, 2008. **13**(11-12): p. 495-501.

3. Sitzmann, M., I.V. Filippov, and M.C. Nicklaus, *Internet resources integrating many small-molecule databases.* SAR QSAR Environ Res, 2008. **19**(1-2): p. 1-9.

4. Kutzler, F.W., et al., *Charge-Density and Bonding in (5,10,15,20-Tetramethylporphyrinato)Nickel(Ii) - a Combined Experimental and Theoretical-Study.* Journal of the American Chemical Society, 1983. **105**(10): p. 2996-3004.

5. Stimpson, D.I. and J.R. Cann, *A Combined Theoretical and Experimental-Study of the Interaction of Metrizamide with Proteins.* Archives of Biochemistry and Biophysics, 1981. **211**(1): p. 403-412.

6. Cripps, S.C., R.S. Orton, and J.E. Carroll, *Combined Theoretical and Experimental Studies of a Push-Pull Trapatt Circuit.* International Journal of Electronics, 1974. **37**(1): p. 1-21.

7. Gaulton, A., et al., *ChEMBL: a large-scale bioactivity database for drug discovery.* Nucleic Acids Res, 2012. **40**(Database issue): p. D1100-7.

8. Izgi, T., et al., *FT-IR and NMR investigation of 2-(1-cyclohexenyl)ethylamine: A combined experimental and theoretical study.* Spectrochimica Acta Part a-Molecular and Biomolecular Spectroscopy, 2007. **68**(1): p. 55-62.

9. de Matos, P., et al., *Chemical Entities of Biological Interest: an update.* Nucleic Acids Research, 2010. **38**: p. D249-D254.

10. Kwan, E.E. and R.Y. Liu, *Enhancing NMR Prediction for Organic Compounds Using Molecular Dynamics.* Journal of Chemical Theory and Computation, 2015. **11**(11): p. 5083-5089.

11. Knox, C., et al., *DrugBank 3.0: a comprehensive resource for 'Omics' research on drugs.* Nucleic Acids Research, 2011. **39**: p. D1035-D1041.

12. Ulrich, E.L., et al., *BioMagResBank.* Nucleic Acids Research, 2008. **36**: p. D402-D408.

13. Wishart, D.S., et al., *HMDB: a knowledgebase for the human metabolome.* Nucleic Acids Res, 2009. **37**(Database issue): p. D603-10.

14. Fourches, D., E. Muratov, and A. Tropsha, *Trust, But Verify: On the Importance of Chemical Structure Curation in Cheminformatics and QSAR Modeling Research.* Journal of Chemical Information and Modeling, 2010. **50**(7): p. 1189-1204.

15. Williams, A.J., S. Ekins, and V. Tkachenko, *Towards a gold standard: regarding quality in public domain chemistry databases and approaches to improving the situation.* Drug Discovery Today, 2012. **17**(13-14): p. 685-701.

16. Warr, W.A., *Many InChIs and quite some feat.* Journal of Computer-Aided Molecular Design, 2015. **29**(8): p. 681-694.

17. Dalby, A., et al., *Description of Several Chemical-Structure File Formats Used by Computer-Programs Developed at Molecular Design Limited.* Journal of Chemical Information and Computer Sciences, 1992. **32**(3): p. 244-255.

18. Sarotti, A.M. and S.C. Pellegrinet, *Application of the Multi-standard Methodology for Calculating H-1 NMR Chemical Shifts.* Journal of Organic Chemistry, 2012. **77**(14): p. 6059-6065.

19. Weininger, D., *Smiles, a Chemical Language and Information-System .1. Introduction to Methodology and Encoding Rules.* Journal of Chemical Information and Computer Sciences, 1988. **28**(1): p. 31-36.

20. O'Boyle, N.M., *Towards a Universal SMILES representation - A standard method to generate canonical SMILES based on the InChI.* J Cheminform, 2012. **4**(1): p. 22.

21. Yang, J., S.X. Huang, and Q.S. Zhao, *Structure Revision of Hassananes with Use of Quantum Mechanical (13)C NMR Chemical Shifts and UV-Vis Absorption Spectra.* Journal of Physical Chemistry A, 2008. **112**(47): p. 12132-12139.

22. Seca, A.M.L., et al., *Chemical composition of the light petroleum extract of Hibiscus cannabinus bark and core.* Phytochemical Analysis, 2000. **11**(6): p. 345-350.

23. Watts, H.D., M.N.A. Mohamed, and J.D. Kubicki, *Comparison of Multistandard and TMS-Standard Calculated NMR Shifts for Coniferyl Alcohol and Application of the Multistandard Method to Lignin Dimers.* Journal of Physical Chemistry B, 2011. **115**(9): p. 1958-1970.

24. Akhondi, S.A., J.A. Kors, and S. Muresan, *Consistency of systematic chemical identifiers within and between small-molecule databases.* Journal of Cheminformatics, 2012. **4**.

25. Heller, S., et al., *InChI - the worldwide chemical structure identifier standard.* J Cheminform, 2013. **5**(1): p. 7.

26. Heller, S.R., et al., *InChI, the IUPAC International Chemical Identifier.* Journal of Cheminformatics, 2015. **7**.

27. Southan, C., *InChI in the wild: an assessment of InChIKey searching in Google.* Journal of Cheminformatics, 2013. **5**.

28. Pletnev, I., et al., *InChIKey collision resistance: an experimental testing.* J Cheminform, 2012. **4**(1): p. 39.

29. Alver, O., *DFT, FT-Raman, FT-IR, solution and solid state NMR studies of 2,4-dimethoxyphenylboronic acid.* Comptes Rendus Chimie, 2011. **14**(5): p. 446-455.

30. Asiri, A.M., et al., *Synthesis, molecular conformation, vibrational and electronic transition, isometric chemical shift, polarizability and hyperpolarizability analysis of 3-(4-Methoxy-phenyl)-2-(4-nitro-phenyl)-acrylonitrile: A combined experimental and theoretical analysis.* Spectrochimica Acta Part a-Molecular and Biomolecular Spectroscopy, 2011. **82**(1): p. 444-455.

31. Bagno, A., F. Rastrelli, and G. Saielli, *Predicting the NMR spectra of nucleotides by DFT calculations: cyclic uridine monophosphate.* Magnetic Resonance in Chemistry, 2008. **46**(6): p. 518-524.

32. Borkowski, E.J., F.D. Suvire, and R.D. Enriz, *Advances in correlation between experimental and DFT/GIAO computed C-13 NMR chemical shifts: A theoretical study on pentacyclic terpenoids (fernenes).* Journal of Molecular Structure-Theochem, 2010. **953**(1-3): p. 83-90.

33. Coruh, A., et al., *Synthesis, molecular conformation, vibrational, electronic transition, and chemical shift assignments of 4-(thiophene-3-ylmethoxy)phthalonitrile: a combined experimental and theoretical analysis.* Structural Chemistry, 2011. **22**(1): p. 45-56.

34. Fulmer, G.R., et al., *NMR Chemical Shifts of Trace Impurities: Common Laboratory Solvents, Organics, and Gases in Deuterated Solvents Relevant to the Organometallic Chemist.* Organometallics, 2010. **29**(9): p. 2176-2179.

35. Hill, D.E., N. Vasdev, and J.P. Holland, *Evaluating the accuracy of density functional theory for calculating H-1 and C-13 NMR chemical shifts in drug molecules.* Computational and Theoretical Chemistry, 2015. **1051**: p. 161-172.

36. Karabacak, M., et al., *Experimental (UV, NMR, IR and Raman) and theoretical spectroscopic properties of 2-chloro-6-methylaniline.* Molecular Physics, 2009. **107**(3): p. 253-264.

37. Krishnakumar, V., D. Barathi, and R. Mathammal, *Molecular structure, vibrational spectra, HOMO, LUMO and NMR studies of 1,2-dichloro-4-nitrobenzene and 2,3,5,6-tetrachloro-1-nitrobenzene based on density functional calculations.* Spectrochimica Acta Part a-Molecular and Biomolecular Spectroscopy, 2012. **86**: p. 196-204.

38. Krishnakumar, V., et al., *Molecular structure, vibrational spectra, HOMO, LUMO and NMR studies of 2-chloro-4-nitrotoluene and 4-chloro-2-nitrotoluene.* Spectrochimica Acta Part a-Molecular and Biomolecular Spectroscopy, 2012. **91**: p. 1-10.

39. Krishnakumar, V., et al., *Molecular structure, spectroscopic studies (FTIR, FT-Raman and NMR) and HOMO-LUMO analysis of 6-chloro-o-cresol and 4-chloro-3-methyl phenol by density functional theoretical study.* Spectrochimica Acta Part a-Molecular and Biomolecular Spectroscopy, 2012. **97**: p. 144-154.

40. Li, Y.J., et al., *Screening and characterization of natural antioxidants in four Glycyrrhiza species by liquid chromatography coupled with electrospray ionization quadrupole time-of-flight tandem mass spectrometry.* Journal of Chromatography A, 2011. **1218**(45): p. 8181-8191.

41. Lomas, J.S., *H-1 NMR spectra of alcohols in hydrogen bonding solvents: DFT/GIAO calculations of chemical shifts.* Magnetic Resonance in Chemistry, 2016. **54**(1): p. 28-38.

42. Osmialowski, B., E. Kolehmainen, and R. Gawinecki, *GIAO/DFT calculated chemical shifts of tautomeric species. 2-Phenacylpyridines and (Z)-2-(2-hydroxy-2-phenylvinyl)pyridines.* Magnetic Resonance in Chemistry, 2001. **39**(6): p. 334-340.

43. Parlak, C., et al., *Molecular structure, NMR analyses, density functional theory and ab initio Hartree-Fock calculations of 4,4 '-diaminooctafluorobiphenyl.* Journal of Molecular Structure, 2008. **891**(1-3): p. 151-156.

44. Perez, M., et al., *Accuracy vs time dilemma on the prediction of NMR chemical shifts: A case study (chloropyrimidines).* Journal of Organic Chemistry, 2006. **71**(8): p. 3103-3110.

45. Rablen, P.R., S.A. Pearlman, and J. Finkbiner, *A comparison of density functional methods for the estimation of proton chemical shifts with chemical accuracy.* Journal of Physical Chemistry A, 1999. **103**(36): p. 7357-7363.

46. Sarotti, A.M. and S.C. Pellegrinet, *A Multi-standard Approach for GIAO C-13 NMR Calculations.* Journal of Organic Chemistry, 2009. **74**(19): p. 7254-7260.

47. Sebastian, S., et al., *Quantum mechanical study of the structure and spectroscopic (FT-IR, FT-Raman, C-13, H-1 and UV), first order hyperpolarizabilities, NBO and TD-DFT analysis of the 4-methyl-2-cyanobiphenyl.* Spectrochimica Acta Part a-Molecular and Biomolecular Spectroscopy, 2011. **78**(2): p. 590-600.

48. Senyel, M., A. Unal, and O. Alver, *Molecular structure, NMR analyses, density functional theory and ab initio Hartree-Fock calculations of 3-phenylpropylamine.* Comptes Rendus Chimie, 2009. **12**(6-7): p. 808-815.

49. Senyel, M., O. Alver, and C. Parlak, *H-1, C-13, N-15 NMR and (n)J(C, H) coupling constants investigation of 3-piperidino-propylamine: A combined experimental and theoretical study.* Spectrochimica Acta Part a-Molecular and Biomolecular Spectroscopy, 2008. **71**(3): p. 830-834.

50. Sridevi, C., G. Shanthi, and G. Velraj, *Structural, vibrational, electronic, NMR and reactivity analyses of 2-amino-4H-chromene-3-carbonitrile (ACC) by ab initio HF and DFT calculations.* Spectrochimica Acta Part a-Molecular and Biomolecular Spectroscopy, 2012. **89**: p. 46-54.

51. Tormena, C.F. and G.V.J. da Silva, *Chemical shifts calculations on aromatic systems: a comparison of models and basis sets.* Chemical Physics Letters, 2004. **398**(4-6): p. 466-470.

52. Vijaya, P. and K.R. Sankaran, *A combined experimental and DFT study of a novel unsymmetrical azine 2-(4-methoxybenzylidene)-1-(1-(4-isobutylphenyl) ethylidene)hydrazine.* Spectrochimica Acta Part a-Molecular and Biomolecular Spectroscopy, 2015. **138**: p. 460-473.

53. Wiitala, K.W., T.R. Hoye, and C.J. Cramer, *Hybrid density functional methods empirically optimized for the computation of C-13 and H-1 chemical shifts in chloroform solution.* Journal of Chemical Theory and Computation, 2006. **2**(4): p. 1085-1092.

54. Wiitala, K.W., et al., *Evaluation of various DFT protocols for computing H-1 and C-13 chemical shifts to distinguish stereoisomers: diastereomeric 2-, 3-, and 4-methylcyclohexanols as a test set.* Journal of Physical Organic Chemistry, 2007. **20**(5): p. 345-354.

55. Willoughby, P.H., M.J. Jansma, and T.R. Hoye, *A guide to small-molecule structure assignment through computation of (H-1 and C-13) NMR chemical shifts.* Nature Protocols, 2014. **9**(3): p. 643-660.

56. Feunang, Y.D., et al., *ClassyFire: automated chemical classification with a comprehensive, computable taxonomy.* Journal of Cheminformatics, 2016. **8**.

57. Xin, D., et al., *Development of a (13)C NMR Chemical Shift Prediction Procedure Using B3LYP/cc-pVDZ and Empirically Derived Systematic Error Correction Terms: A Computational Small Molecule Structure Elucidation Method.* J Org Chem, 2017. **82**(10): p. 5135-5145.

58. Harris, R.K., et al., *NMR nomenclature. Nuclear spin properties and conventions for chemical shifts - (IUPAC recommendations 2001).* Pure and Applied Chemistry, 2001. **73**(11): p. 1795-1818.

59. Harris, R.K., et al., *Further conventions for NMR shielding and chemical shifts (IUPAC recommendations 2008).* Pure and Applied Chemistry, 2008. **80**(1): p. 59-84.

60. Schuler, R.H., et al., *On the addition of (OH)-O-center dot radicals to the ipso positions of alkyl-substituted aromatics: Production of 4-hydroxy-4-methyl-2,5-cyclohexadien-1-one in the radiolytic oxidation of p-cresol.* Journal of Physical Chemistry A, 2002. **106**(50): p. 12178-12183.

61. Wipf, P. and A.D. Kerekes, *Structure reassignment of the fungal metabolite TAEMC161 as the phytotoxin viridiol.* Journal of Natural Products, 2003. **66**(5): p. 716-718.

62. Timmons, C. and P. Wipf, *Density Functional Theory Calculation of C-13 NMR Shifts of Diazaphenanthrene Alkaloids: Reinvestigation of the Structure of Samoquasine A.* Journal of Organic Chemistry, 2008. **73**(22): p. 9168-9170.

63. Baldridge, K.K. and J.S. Siegel, *Correlation of empirical delta(TMS) and absolute NMR chemical shifts predicted by ab initio computations.* Journal of Physical Chemistry A, 1999. **103**(20): p. 4038-4042.

64. Hoffman, R.E., *Standardization of chemical shifts of TMS and solvent signals in NMR solvents.* Magnetic Resonance in Chemistry, 2006. **44**(6): p. 606-616.

65. Hoffman, R.E., *Variations on the chemical shift of TMS.* Journal of Magnetic Resonance, 2003. **163**(2): p. 325-331.

66. Kohavi, R., *A Study of Cross-Validation and Bootstrap for Accuracy Estimation and Model Selection*, in *International Joint Conference on Articial Intelligence*. 1995.

67. Dubitzky, W., M. Granzow, and D.P. Berrar, *Fundamentals of data mining in genomics and proteomics*. 2007: Springer Science & Business Media.

68. Xu, Q.-S. and Y.-Z. Liang, *Monte Carlo cross validation.* Chemometrics and Intelligent Laboratory Systems, 2001. **56**(1): p. 1-11.

69. Vollhardt, P., Schore, N., *Organic Chemistry Structure and Function*. 6th Edition ed. 2009, New York: W. H. Freeman and Company.

70.  *Spectral Database for Organic Compounds, SDBS*. Available from: <http://sdbs.db.aist.go.jp/>.
