# Supplementary material for: An automated framework for NMR chemical shift calculations of small organic molecules
Source: J Cheminform. 2018 Oct 26;10:52. doi: 10.1186/s13321-018-0305-8 (PMC6755567; doi:10.1186/s13321-018-0305-8)
Supplement: Supplementary file 2 — Additional file 2. Supporting information files. Including tutorial, code, and all other files. [file 13321_2018_305_MOESM2_ESM.zip › isicle_tutorial.zip!/isicle_tutorial/ISiCLE_Tutorial.pdf]

ISiCLE-NMR TUTORIAL

Contents

1.1 Getting Started

1.2 What is needed?

1.3 Preparing input files

1.4 Running a simulation

1.5. Generated files by Isicle

1.5.1 Generated files by ISiCLE

1.5.2 Generated files by NWChem

1.6 Output files

1.6.1 Output files

1.6.2 3D Mol files

1.6.3 Excel files

APPENDIX

A1. How to Install Python Bindings

A.1.1 How to Install Openbabel

A.1.2 How to Install Fabric

Introduction

This tutorial provides a first introduction to ISiCLE-NMR chemical shift calculations by using NWChem capabilities. It can also be used as a refresher course for NMR chemical shift calculations for the non-expert NWChem user.

It is assumed Openbabel has been installed correctly on your computer. For installation instructions, please refer to the Openbabel web site: http://openbabel.org/

This tutorial is subdivided in three sections. The first one gives brief descriptions of all files needed for the simulations and covers the preparation of them. The second section describes the execution procedure of ISiCLE. The last section explains the content of output files.

For a detailed description of NWChem, the reader is referred to the NWChem User's guide located at http://www.nwchem-sw.org/.

The examples in the tutorial will focus on the density functional theory (DFT) study of a small set of molecules using two different methods. Our set consists of 3 different small organic molecules: methanol, methyl isothiocyanate and nitromethane. These molecules were chosen to show different cases, because their NMR spectra are well known and their simulations do not take much time.

In this tutorial, the isotropic shielding values will be computed employing two widely used methods, blyp and b3lyp, and two basis sets, 6-31G(d) and 3-21G. Molecular geometries will be optimized by using b3lyp/6-31G(d) and bhlyp/cc-pVDZ. One of the methods are held in gas phase while the other method is used considering a solvent: dimethyl sulfoxide (DMSO). The COSMO solvation model is used to describe dielectric screening effects in solvents.

Table 1. List of molecules that will be used in the simulations.

Molecule name	Molecule formula	InChI codes	InChI Keys
Methanol	CH_3OH	InChI=1S/CH4O/c1-2/h2H,1H3	OKKJLVBELUTLKV-UHFFFAOYSA-N
Methyl isothiocyanate	C_2H_3NS	InChI=1S/C2H3NS/c1-3-2-4/h1H3	LGDSHSYDSCRFAB-UHFFFAOYSA-N
Nitromethane	CH_3NO_2	InChI=1S/CH3NO2/c1-2(3)4/h1H3	LYGJENNIWJXYER-UHFFFAOYSA-N

1.1 Getting Started

Operating System

ISiCLE must be run from the MS-DOS command line in Microsoft Windows.

1.1.1 Required programs

- **Python** must be installed in a user directory with the standard library and may needed to be added the install directory to the PATH. For more information and installation instructions, please visit https://www.python.org/.
- **A text editor** (such as WordPad, Notepad or another text editor) of user's choosing is used for input MOL, XYZ and NWChem files and to view the results in mol files.
- **Microsoft Excel** Microsoft Excel will be used for input files and view the results from ISiCLE simulations.

1.1.2 Required Python Libraries

Openbabel is a chemical toolbox and required unless user supplies only XYZ and MOL files. It is available for Windows at http://openbabel.org/. Installation procedure for OpenBabel is described in Appendix A1.1.

Fabric is a command-line tool for streamlining the use of SSH and required to remotely execute, monitor and control simulations on a server. Installation procedure for Fabric is described in Appendix A1.2.

Others

Other libraries are hashlib, fabric, xlrd, xlswriter shutil, argparse, getpass, os, sys, time, re, glob, math and numpy, all of which can be downloaded using pip.

1.2 What Is Needed?

In order to run ISiCLE, you need the appropriate input files. The files for this tutorial are available in the zipped file called ISiCLE -tutorial. Unzip it in a convenient location. You will see two sub folders named “input-example” and “output example”. The folder “input-example” has many files that ISiCLE will use. Outputs of the simulation is provided in “output-example” subdirectory. If you are not able to simulate the tutorial files, correct versions have been provided and found in the “output-example” folder.

In order to run a simulation via ISiCLE, an InChI code or XYZ file each molecule is required. Also, MOL files can be provided by the user if desired, though this is not required. Each of these options are described in detail below.

1.2.1 InChI Codes

InChI codes are human-readable, non-proprietary identifiers that fully describe molecular structure. They can be found by searching database sites such as PubChem (https://pubchem.ncbi.nlm.nih.gov/) or can be created by the user using a variety of methods. For more information about InChI codes, please visit www.iupac.org. Note that the InChI codes provided by user should be valid and be readable by Openbabel, otherwise ISiCLE will raise an error message.

1.2.2 XYZ Files

XYZ files store atomic symbols and coordinates and can be generated using software such as ChemAxon (https://www.chemaxon.com/) and VMD (www.ks.uiuc.edu/Research/vmd/). A view of the XYZ file of carbon dioxide is as below:

```
3

C          0.97588          0.08066          0.05396
O         -0.22112          0.08066          0.05396
O          2.17288          0.08066          0.05396
```

Figure 1. XYZ file of Carbon dioxide.

1.2.3 MOL Files

MOL files store atomic symbols and coordinates as well as structural information of the molecules, such as bonding interactions. Users can also append experimental chemical shifts to this file type. A view of the 3D MOL file of carbon dioxide (generated using Openbabel) is below:

```
OpenBabel01311706113D

  3  2  0  0  0  0  0  0  0  0999 V2000
    0.9759    0.0807    0.0540 C   0  0  0  0  0  0  0  0  0  0  0  0
   -0.2211    0.0807    0.0540 O   0  0  0  0  0  0  0  0  0  0  0  0
    2.1729    0.0807    0.0540 O   0  0  0  0  0  0  0  0  0  0  0  0
  1  2  2  0  0  0  0
  1  3  2  0  0  0  0
M  END
```

Figure 2. 3D mol file of Carbon dioxide.

1.2.4 NWChem Input Files (optional)

NWChem input files are composed of commands which define various data including basis sets, geometries, filenames and directives to be performed on the given data. ISiCLE generates NWChem input files itself by placing the required inputs given by user. However, the user needs to supply input for each setting that differs from the default values, otherwise these will be used automatically. Since the input file is a free format text file and case insensitive (except for actual data), the user can prepare their own input files. For more information about the input format along with the syntax of NWChem input files, the reader is referred to the NWChem user's guide.

ISiCLE uses an NWChem input file temporarily as in Figure 4.a. It includes some prefixes which are later replaced by ISiCLE based on the option that user enters as in Figure 4.b. The items that are replaced by ISiCLE are numbered, labeled and explained below.

1. Title of the job and directive of "start". In ISiCLE, it includes the InChIKey of the molecule, NMR and geometry methods to be used, nuclei whose NMR chemical shifts are to be looked for and the solvent used. Since the calculations are performed in gas phase in Figure *3*.a, the solvent is not stated.
2. Directory where input files are stored.

3. Geometry file specified with its InChIKey and its full directory. Not that NWChem takes the geometry files only in XYZ format.
4. Basis set and exchange-correlation functional, respectively, used in the optimization of molecular geometry. Note that here the geometry is optimized with the method of b3lyp/6-31G* and these do not have to be the same with yours. *Note: Since ISiCLE uses NWChem for DFT calculations, it is designed to read input variables in the same way as NWChem. For this reason, the user is required to specify a supported functional and basis set.*
5. Base file name of the geometry files at each iteration. It has the InChIKey of the molecule followed by the functional and basis set used in geometry optimization procedure. NWChem automatically writes these geometry files in XYZ file format with the iteration number appended at the end of the base file name.
6. Basis set and exchange-correlation functional, respectively, used in the calculation of the NMR property are stated, respectively. Note that here the isotropic shieldings are calculated with the method of blyp/6-31G* and these do not have to be the same with yours.

The number of atoms whose NMR isotropic shieldings will be calculated along with the sequence numbers of each found in their geometry file. If nothing is specified (as shown in Figure *3*.a), isotropic shieldings of all atoms will be calculated.

1.3 Preparing input files

NWChem has an ability to calculate the isotropic shieldings of certain nuclei. The user needs to enter the atomic symbol of a nucleus which isotropic shieldings will be calculated. He/she can specify multiple nucleus or nuclei in any order as long as written consecutively. The user also needs to provide an InChI code of the reference molecule or the name of its XYZ file. If the reference molecule does not have the nucleus given, ISiCLE raises an error message.

The only files the user needs to prepare are the Excel files: one containing the list of molecules (e.g. MoleculeSet.xlsx in the tutorial) and the other one containing all options specified by the user (e.g. Methods.xlsx in the tutorial).

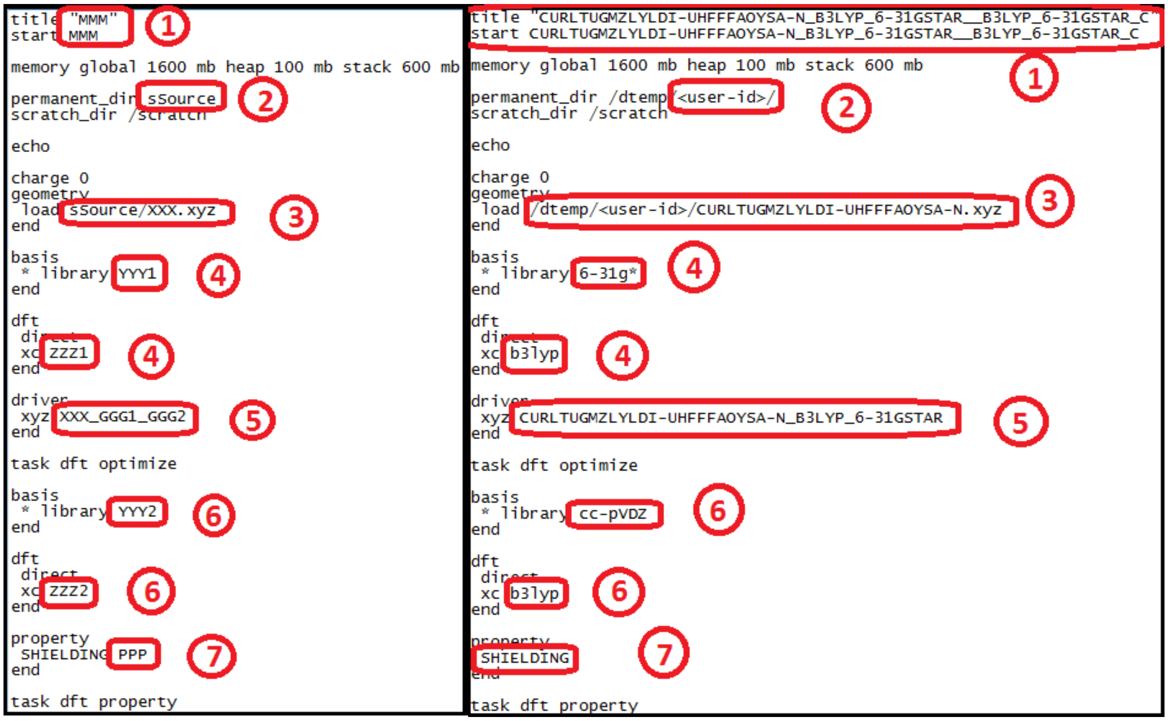

Figure 3. a) File used as a base NWChem input file b) Example NWChem input file prepared by ISiCLE.

• **<u>Excel file for Molecule Set</u>:** An Excel sheet listing InChI codes of each molecule. InChI codes should be written in the first column starting from the first row of the sheet as below.

• **<u>Excel file for Methods</u>:** An Excel sheet with DFT simulation options which will be adopted by NWChem and ISiCLE in running the simulation.

Each should be written in order as below:

1st column: Functional used for isotropic shielding calculations

2nd column: Basis set used for isotropic shielding calculations

3rd column: Functional used for geometry optimizations

4th column: Basis set used for geometry optimizations

5th column: Nuclei whose isotropic shieldings are calculated

6th column: Solvents in which NMR calculations are performed

7th column: InChI codes of reference molecules

	A
1	InChI=1S/CH3NO2/c1-2(3)4/h1H3
2	InChI=1S/CH4O/c1-2/h2H,1H3
3	LGDSHSYDSCRFAB-UHFFFAOYSA-N.xyz

Figure 4. Screen shot of an Excel sheet which lists the molecules used in tutorial simulations.

Isotropic shieldings are converted to chemical shift values by subtracting the isotropic shielding constants for the specified nuclei in the molecule of interest from those of reference compound computed at the same level of theory as in the following equation (Eqn 1) below.

$$\delta_i = \sigma_{ref} - \sigma_i + \delta_{ref} \tag{1}$$

where δ_i is chemical shift of atom *i* of the molecule of interest, σ_{ref} is isotropic shielding constant of reference molecule, σ_i is isotropic shielding constant of atom *i* chemical shift of the molecule of interest, and δ_{ref} is chemical shift of reference molecule.

Any molecule can be used as reference in ISiCLE as long as it has the specified nuclei and its experimental chemical shifts are supplied by user unless the reference compound is TMS. Since the experimental chemical shifts of TMS are considered as zero, the calculation of NMR chemical shifts needs only isotropic shieldings of TMS.

8th column: Scaling option for chemical shifts.

Linear regression approach is used for the data of chemical shifts vs. experimental data. If user enters "yes", it scales chemical shifts by linear regression if experimental data of at least two molecules are given. Otherwise it leaves chemical shifts as they are. The following equation is used to find the slope and intercept values:

$$\delta_{experimental} = \frac{intercept - \delta_{calculated}}{-slope} \tag{2}$$

where $\delta_{calculated}$ is the list of calculated chemical shits of molecules with Eqn 1 and $\delta_{experimental}$ is the list of experimental chemical shifts.

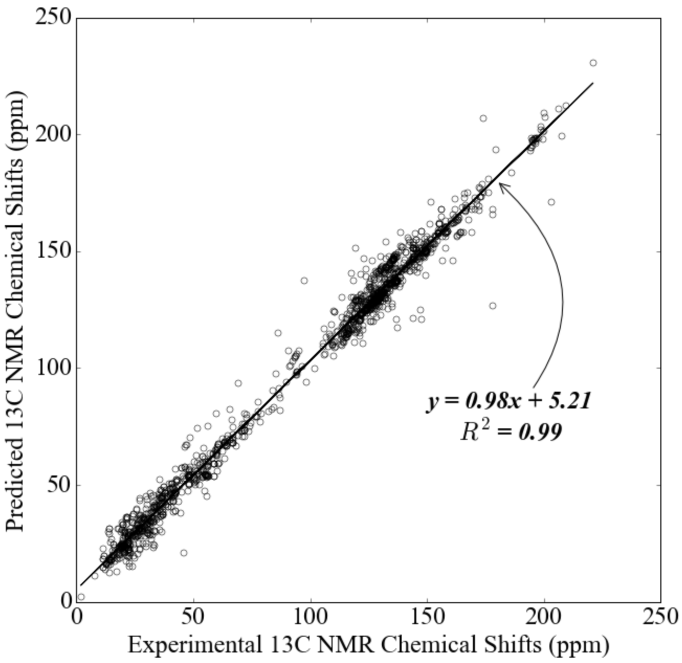

Figure 5. Computed ^{13}C chemical shifts vs. experimental chemical shifts. Plot is taken from the paper and chemical shifts are calculated using the DFT method: GIAO/B3LYP/cc-pvDZ//B3LYP/6-31+G(d).

Alternatively, user can enter his/her own slope and intercept values for each nucleus separated by comma to scale the chemical shifts. For the inputs other than those, scaling will not be performed. 9th column: Scaling option for chemical shifts.

Linear regression approach is used for the data of isotropic shieldings vs. experimental data. If user enters "yes", it scales chemical shifts by linear regression if experimental data of at least two molecules are given. Otherwise it leaves isotropic shieldings as they are. Alternatively, user can enter his/her own slope and intercept values for each nucleus separated by comma to scale the isotropic shieldings. For the inputs other than those, scaling will not be performed. Slope and intercept values are found from equation 3.

$$\delta_{experimental} = \frac{intercept - \sigma_{calculated}}{-slope} \quad (3)$$

where σ is the list of isotopic shielding constants of molecules and δ is the list of experimental chemical shifts.

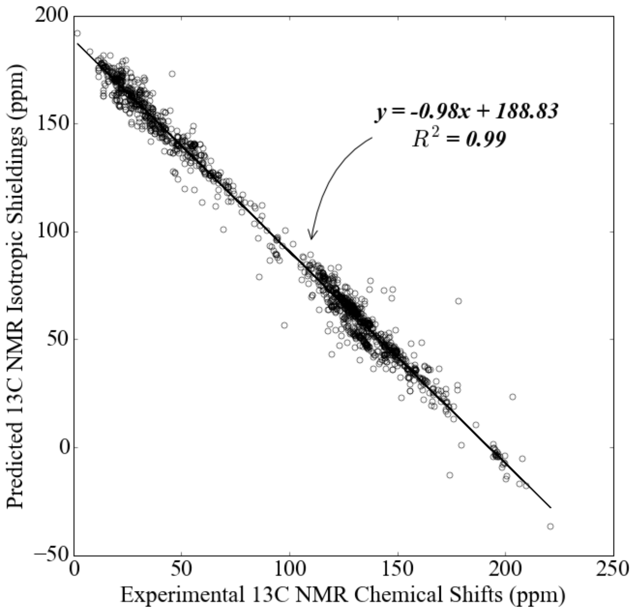

Figure 6. Computed ^{13}C isotropic shieldings vs. experimental chemical shifts. Plot is taken from the paper and chemical shifts are calculated using the DFT method: GIAO/B3LYP/cc-pvDZ//B3LYP/6-31+G(d).

	A	B	C	D
1	NMR functional	NMR basis set	Geometry fucntional	Geometry basis set
2	becke88 lyp	6-31g*	slater 0.8 becke88 nonlocal 0.72 HFexch 0.2	6-31g*
3	b3lyp	3-21g	bhlyp	cc-pVDZ

	E	F	G	H	I
1	Nucleus	Solvent	Reference	ScalingShift	ScalingShielding
2	C	gas	InChI=1S/C4H12Si/c1-5(2,3)4/h1-4H3	no	no
3	CH	dmso	CZDYPVPMEAXLPK-UHFFFAOYSA-N.xyz	yes	yes

Figure 7. Screen shot of an Excel sheet specifying DFT methods used in simulations.

As shown in Figure 6, each item should be properly placed in its own cell in the required order. User is asked to be careful when typing variables into their Excel files and be sure that they are correct before submitting a simulation to be run. It is important to run it correctly. A typographical error or misplacing variables will simply cause the simulation not to run.

In the Figure 6, there are three different simulations are specified. In the first method, the widely used method, b3lyp, is used with the basis set 6-31G(d) for geometry optimization and blyp is used used with the basis set 3-11G for isotropic shieldings and. You may realize that b3lyp is entered

as “slater 0.8 becke88 nonlocal 0.72 HFexch 0.2” in the third column and blyp is specified as “becke88 lyp” in NWChem. It is to show that user can specify any combination of the supported correlation functional options can be used. Isotropic shieldings will be calculated for only Carbon atom as specified and there is no solvent used for this method. NMR chemical shifts will be calculated relative to tetramethylsilane whose InChI code is given and they will not be scaled as not specified.

In the second one, isotropic shieldings are calculated with the method of b3lyp/3-21g//bhlyp/cc-pVDZ in DMSO. What is different here is two nuclei are specified as “CH” and XYZ file of tetramethylsilane is specified instead of its InChI code. Chemical shifts are also calculated by scaling isotropic shieldings on the base of assumption that experimental data is supplemented by user.

1.4 Running a simulation

ISiCLE asks users type the required commands in their MS-DOS command windows. User should use it first to navigate to the directory containing tutorial files.

Specifically, to run the input files in this tutorial, use a Terminal to go to the regarding directory where the tutorial files are located. Make sure that all the required files for a simulation are in that directory. Type the following command

python isicle.py --help

In your screen you will see what arguments ISiCLE requires as in Figure 7.

ISiCLE uses “argparse” module which provides user friendly command line interfaces and makes it easier to user to write the required arguments. ISiCLE automatically generates useful help and usage messages how to enter the inputs. It describes briefly each argument does and how it gets input. It also issues errors when invalid arguments are passed into the program and even does not start the program unless the required arguments are given by the users.

```
usage: isicle.py [-h] [-accnt SACCOUNT] [-molcs SFILENAME1] [-metd SFILENAME2]
                 [-host SHOST] [-dest SSERVERDIR] [-nn INODESNO]
                 [-nc IPROCESSORNOPERNODE] [-time SWALLTIME]

Short sample app

optional arguments:
  -h, --help            show this help message and exit
  -accnt SACCOUNT       Cascade project account, Ex: -accnt cascade12345
  -molcs SFILENAME1     Molecule set with InchI codes, Ex: -molcs
                        Molecule_Set.xlsx
  -metd SFILENAME2      Mol, -metd Ex: Methods.xlsx
  -host SHOST           Cascade account name -host Ex:
                        NetworkID@cascade.emsl.pnl.gov
  -dest SSERVERDIR      File directory in machine, Ex: -dest /dtemp/NetworkID/
  -nn INODESNO          Number of nodes, Ex: -nn 10
  -nc IPROCESSORNOPERNODE
                        Number of processor per node, Ex: 16
  -time SWALLTIME       Maximum running time, Ex: -time 00:10:00
```

Figure 8. Screen shot of the arguments that ISiCLE uses.

Parsers are briefly explained such that:

Parser 1: It gets project account id in server.

In this tutorial, i.e. *<project-id>*

Parser 2: It gets the name of the Excel file which has the molecule set.

In this tutorial, i.e. MoleculeSet.xlsx

Parser 3: It gets the name of the Excel file in which user specifies the simulation options.

In this tutorial, i.e. Method.xlsx

Parser 4: It gets user account name in server

In this tutorial, i.e. <network-id>@cascade.emsl.pnl.gov

Parser 5: It gets the file directory in the server

In this tutorial, i.e. /dtemp/<network-id>/

Parser 6: It gets the number of nodes which will be used in the server. This parser is optional. The default is passed unless user specifies it. Default number of nodes is 1.

In this tutorial, i.e. 6

Parser 7: It gets the number of processors which will be used in the server. This parser is optional. The default is passed unless user specifies it. Default number of processor is 16.

In this tutorial, i.e. 16

Parser 8: It gets the maximum simulation time which will be used in the server. This parser is optional. The default is passed unless user specifies it. Default time value is 5 minutes.

In this tutorial, i.e. 00:15:00

It is time to put all arguments together. Please open a terminal window which executes python for Windows in 32-bit mode. This is due to the fact that Openbabel only works with 32-bit Windows. In a Terminal Window, run your simulation by changing the directory to where the folder "isicle_tutorial_files/input-example" is stored then type the following command:

python isicle.py *–accnt <project_id>* ***-molcs*** *MoleculeSet.xlsx* ***-metd*** *Method.xlsx* ***-host*** *<network-id>@cascade.emsl.pnl.gov* ***-dest*** */dtemp/<network-id>/* ***-nn*** *6* ***-nc*** *16* ***-time*** *00:15:00*

Note that you need to replace the input of network-id with your account id. Your simulation for the tutorial will take about 10-15 minutes to complete, as indicated by setting the time to 15 minutes. NWChem will produce the output files and ISiCLE will perform the rest of the processes. Simulation time may vary depending on the speed of your Internet connection, speed of your computer, etc.

1.5 Generated files

1.5.1 Generated files by ISiCLE

ISiCLE generates the following files in user's computer for each molecule unless already found in that directory.

- 3D MOLfile
- XYZ file
- NWChem input file
- Results Excel file
- Summaries Excel file
- Log file

As mentioned above, user has an option to supply his/her own geometry files and NWChem input files.

1.5.2 Generated files by NWChem

The following files are generated by NWChem for each molecule.

- Output file
- Movecs file

- Hess file
- Data Base file
- Shieldcphf file
- XYZ files

None of these files are in binary code and can be opened with a text editor. The original files should also still be in the directory.

1.6 Output files

1.6.1 NWChem Output file

These files are identified with the specified extension of ".output" and generated automatically by NWChem right after simulation of that molecule starts. It initially states the NWChem input file and takes the geometric coordinates of the molecule. It reports every detail regarding to energy minimization steps and isotropic shieldings of specified atoms. It concludes the total CPU time that takes for that simulation.

ISiCLE monitors each output file in certain time intervals and knows if the simulation is finished by checking the very last sentence of their.

1.6.2 3D MOL files

- **Isotropic shieldings**

ISiCLE exports isotropic shieldings from output files and appends them to 3D mol files. It also writes CPU times and methods used.

- **NMR Chemical shifts**

ISiCLE calculates NMR chemical shifts relative to the given reference molecule and appends them to 3D mol files. It also writes CPU times and methods used. In this tutorial, tetramethylsilane is given as a reference molecule.

- **NMR Chemical shifts scaled by using linear regression on the plot of Calculated shifts vs. Experimental shifts**

ISiCLE scales the NMR chemical shifts calculated relative to the given reference molecule in previous step and appends them to 3D mol files. It also writes slope and intercept values used. This

method is recommended when experimental data of a large molecule set is available. As mentioned above, user has an option to enter slope and intercept values separated by comma for each nucleus to scale NMR chemical shifts.

- **NMR Chemical shifts scaled by using linear regression on the plot of Isotropic shieldings vs. Experimental shifts**

ISiCLE scales the isotropic shieldings extracted from NWChem output files in the first step and appends them to 3D mol files. It also writes slope and intercept values used. This method is recommended when experimental data of a large molecule set is available. As mentioned above, user has an option to enter slope and intercept values separated by comma for each nucleus to scale isotropic shieldings.

1.6.3 Excel files

- **Results.xlsx file**

Results.xlsx file shows the errors for each molecule. Each tab is for each method that user specified. You had 2 different methods in this tutorial.

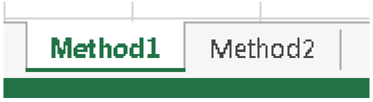

Figure 10 is what you will see in the first sheet of Results.xlsx, which if Method 1. In the first row the method used is written. In the second row, you will see 5 different types of error for ^{13}C. Units are in ppm.

Error types are written below with their formula used:

- Mean Absolute Error: It measures how close the calculated shifts are to the experimental chemical shifts.

$$MAE = \frac{1}{n}\sum_{i=1}^{n}\left|\delta_{calc,i} - \delta_{exp,i}\right|$$

- Mean Squared Error: It measures the average of the squares of the errors in calculated chemical shifts.

$$MSE = \frac{1}{n}\sum_{i=1}^{n}(\delta_{calc,i} - \delta_{exp,i})^2$$

- Root Mean Square Error: It measures the difference between the calculated chemical shifts and experimental chemical shifts.

$$RMSE = \sqrt{\frac{\sum_{i=1}^{n}(\delta_{calc,i} - \delta_{exp,i})^2}{n}}$$

```
 OpenBabel10251615103D

  6  5  0  0  0  0  0  0  0  0999 V2000
    0.9916   -0.0344    0.0943 C   0  0  0  0  0  0  0  0  0  0  0  0
    2.4072   -0.0203    0.0914 O   0  0  0  0  0  0  0  0  0  0  0  0
    0.6314   -0.3536    1.0754 H   0  0  0  0  0  0  0  0  0  0  0  0
    0.6314   -0.7184   -0.6781 H   0  0  0  0  0  0  0  0  0  0  0  0
    0.6258    0.9735   -0.1154 H   0  0  0  0  0  0  0  0  0  0  0  0
    2.7026   -0.9272    0.2801 H   0  0  0  0  0  0  0  0  0  0  0  0
  1  2  1  0  0  0  0
  1  3  1  0  0  0  0
  1  4  1  0  0  0  0
  1  5  1  0  0  0  0
  2  6  1  0  0  0  0
M  END

Experimental Shifts
FT spectra:     Speed:100.40 MHz for 13C & 399.65 MHz for 1H    Spectra resoultion:     Solvent:0.010
g/0.5 ml CDCl3          Temperature:    Reference: http://sdbs.db.aist.go.jp/sdbs/cgi-bin/cre_index.cgi
1       C       50.74
2       O
3       H       3.474
4       H       3.474
5       H       3.474
6       H       1.67


Isotropic Shielding
B3LYP/3-21G//BHLYP/CC-PVDZ/CH/DMSO
Number of nodes: 0      Number of cores per node: 0     Total CPU time: 5.1s    Total time: 5.1s
1  C  156.5069
3  H  29.3068
4  H  29.3068
5  H  29.3171
6  H  33.9147


Calculated Shifts Relative to the Reference of: CZDYPVPMEAXLPK-UHFFFAOYSA-N
B3LYP/3-21G//BHLYP/CC-PVDZ/CH/DMSO
Number of nodes: 0      Number of cores per node: 0     Total CPU time: 5.1s    Total time: 5.1s
1       C       48.3501
3       H       3.7614
4       H       3.7614
5       H       3.7511
6       H       -0.8465


Scaled Shifts Relative to the Reference of: CZDYPVPMEAXLPK-UHFFFAOYSA-N
B3LYP/3-21G//BHLYP/CC-PVDZ/CH/DMSO
Number of nodes: 0      Number of cores per node: 0     Total CPU time: 5.1s    Total time: 5.1s
Slope: [0.7637660241030401, 2.040440264594427]  Intercept: [8.448816891715005, -3.4852648884618427]
R_squared: [0.9921320881016068, 0.90816399137903936]
1       C       52.2428
3       H       3.5515
4       H       3.5515
5       H       3.5465
6       H       1.2932


Predicted Shifts by Linear Regression
B3LYP/3-21G//BHLYP/CC-PVDZ/CH/DMSO
Number of nodes: 0      Number of cores per node: 0     Total CPU time: 5.1s    Total time: 5.1s
Slope: [-0.7637660241030391, -2.0404402645944275]       Intercept: [196.408183108285, 36.55346488846184]
R_Squared: [0.9921320881016068, 0.90816399137903936]
1       C       52.2428
3       H       3.5515
4       H       3.5515
5       H       3.5465
6       H       1.2932
```

Figure 9. Screen shot of the 3D mol file of Hydrogen cyanide after running with Method 2 in the tutorial.

- Mean Signed Error: It assesses how well the calculated chemical shifts match the experimental shift values.

$$MSE = \sum_{i=1}^{n} \frac{(\delta_{calc,i} - \delta_{exp,i})}{n}$$

- Maximum Absolute Error: It shows how much a nucleus is far away from the value that is supposed to be.

$$S = \{(\delta_{calc,i} - \delta_{exp,i})\}$$

where $\delta_{calc,i}$ is chemical shift of a molecule, $\delta_{exp,i}$ is its experimental shift and S is the cluster of absolute errors of its each element. The maximum absolute error is the element of the set that is greater than every other element of S.

	A	B	C	D	E	F	G	H
1	BECKE88_LYP_6-31GSTAR__SLATER_0.8_BECKE88_NONLOCAL_0.72_HFEXCH_0.2_6-3							
2	InChI	InChIKey	Mean Abs	Mean Squ	Root Mea	Mean Sigr	Maximum	CPU time (s)
3	Calculated Shifts							
4	InChI=1S/	LYGJENNI	1.4785	2.1859622	1.4785	-1.4785	1.4785	5.2
5	InChI=1S/	OKKJLVBE	0.4365	0.1905322	0.4365	0.4365	0.4365	1.6
6		LGDSHSYD	3.94945	29.010962	5.3861825	-3.94945	7.6118	12.7

Figure 10. Screen shot of the second sheet of Results.xlsx file.

Figure 11 shows you the screen shot of the second method. Here in this method two different nuclei is specified: ^{13}C and ^{1}H. The same type of errors are calculated for each of them. Since scaled shifts are also asked in this method, their errors are reported for between 7th and 14th rows.

- **Summaries.xlsx file**

This file shows the average errors for the set of molecules for each method. Since you had 2 different methods in this tutorial, you will see three rows (A4:A6) which lists the methods used as in Figure 12.

	A	B	C	D	E	F	G	H	I	J	K	L	M
1	B3LYP_3-21G BHLYP_CC-PVDZ_CH_DMSO												
2	InChI	InChIKey	Mean Abs	Mean Squ	Root Mea	Mean Sigr	Maximum	Mean Abs	Mean Squ	Root Mea	Mean Sigr	Maximum	CPU time (s)
3	Calculated Shifts												
4	InChI=1S/	LYGJENNI	2.9171	8.5094724	2.9171	-2.9171	2.9171	0.5911666	0.4220077	0.6496212	0.5911666	0.7816	16.9
5	InChI=1S/	OKKJLVBE	2.3899	5.7116220	2.3899	-2.3899	2.3899	0.8421	1.6436885	1.2820641	-0.41615	2.5165	5.1
6		LGDSHSYD	12.66055	274.63512	16.572118	-12.66055	23.3538	0.5158333	0.2663274	0.5160692	0.5158333	0.5379	72.9
7	Scaled Shifts Slope[0.7637660241030401, 2.040440264594427] Intercept[8.448816891715005, -3.4852648884618427] Corr Coeff[0.9921320881016068, 0.90816399137903936]												
8	InChI=1S/	LYGJENNI	4.4778	20.050692	4.4778	4.4778	4.4778	0.2157333	0.0639630	0.2529092	-0.215733	0.4024	
9	InChI=1S/	OKKJLVBE	1.5028	2.2584078	1.5028	1.5028	1.5028	0.151075	0.0398117	0.1995288	-0.037325	0.3768	
10		LGDSHSYD	2.9903	10.430294	3.2295965	-2.9903	4.2103	0.2654333	0.0705138	0.2655445	0.2654333	0.2763	
11	Predicted Shifts by Linear Regression Slope[-0.7637660241030391, -2.0404402645944275] Intercept[196.408183108285, 36.55346488846184] Corr Coeff[0.9921320881016068, 0.90816399137903936]												
12	InChI=1S/	LYGJENNI	4.4778	20.050692	4.4778	4.4778	4.4778	0.2157333	0.0639630	0.2529092	-0.215733	0.4024	
13	InChI=1S/	OKKJLVBE	1.5028	2.2584078	1.5028	1.5028	1.5028	0.151075	0.0398117	0.1995288	-0.037325	0.3768	
14		LGDSHSYD	2.9903	10.430294	3.2295965	-2.9903	4.2103	0.2654333	0.0705138	0.2655445	0.2654333	0.2763	

Figure 11. Screen shot of the second sheet of Results.xlsx file.

	A	B	C	D	E	F	G
1	Methods	Errors					
2		Errors of Shifts Calculated Relative to the Reference of CZD					
3		Mean Abs	Root Mea	Maximum	Mean Abs	Root Mea	Maximum
4	BECKE88_	1.954817	2.433728	3.1756			
5	B3LYP/3-2	5.989183	7.29304	9.5536	0.6497	0.815918	1.278667

	H	I	J	K	L	M	N	O	P	Q	R	S
1	Errors of Scaled Shifts Relative to the Reference of CZDYPVPMEAXLPK-UHFFFAOYSA-N.xyz											
2	Mean Abs	Root Mea	Maximum	Slope for	Intercept	R_Square	Mean Abs	Root Mea	Maximum	Slope for	Intercept	R_Square
3												
4	2.9903	3.070066	3.396967	0.7637660	8.4488168	0.9921320	0.210747	0.239328	0.351833	2.040440	-3.485264	0.908163
5												

	T	U	V	W	X	Y	Z	AA	AB	AC	AD	AE
1												
2	Errors of Scaled Shieldings											
3	Mean Abs	Root Mea	Maximum	Slope for	Intercept	R_Square	Mean Abs	Root Mea	Maximum	Slope for	Intercept	R_Square
4												
5	2.9903	3.070066	3.396967	-0.763766	196.40818	0.9921320	0.210747	0.239328	0.351833	-2.040440	36.55346	0.908163

	AF	AG	AH	AI
1				
2				
3	Average C	Standard Deviation of CPU		
4	6.5	4.623851		
5	31.63333	29.57491		

Figure 12. Screen shot of the Summaries.xlsx file.

APPENDIX

A1. How to Install Python Bindings

A.1.1 How to Install Openbabel

Please follow the steps below:

1. Download the OpenBabel from the following website:
http://openbabel.org/wiki/Category:Installation

2. The file you downloaded is an executable file, double click the Openbabel icon to start the setup process.

3. Download Openbabel Python bindings for your Python version.

Python 2.5	https://sourceforge.net/projects/openbabel/files/openbabel-python/1.7/openbabel-python-1.7.py25.exe/download
Python 2.6	https://sourceforge.net/projects/openbabel/files/openbabel-python/1.7/openbabel-python-1.7.py26.exe/download
Python 2.7	https://sourceforge.net/projects/openbabel/files/openbabel-python/1.7/openbabel-python-1.7.py27.exe/download
Python 3.1	https://sourceforge.net/projects/openbabel/files/openbabel-python/1.7/openbabel-python-1.7.py31.exe/download
Python 3.2	https://sourceforge.net/projects/openbabel/files/openbabel-python/1.7/openbabel-python-1.7.py32.exe/download

4. Install the Openbabel Python binding that you downloaded.

Note: Openbabel should be in the folder named BABEL_DATADIR. Add "BABEL_DATADIR" to the environment variables of your system, if necessary.

You may find the necessary commands at:
http://openbabel.org/docs/current/UseTheLibrary/PythonInstall.html

A.1.2 How to Install Fabric

Please follow the steps below:

1. Download the Fabric in a tar.gz or zip archive from the following website:
https://pypi.python.org/pypi/Fabric

2. Make sure you have setuptools packages in your python. If you don't have, follow the link below:

https://pypi.python.org/pypi/setuptools

3. Make sure you have Paramiko SSH library. If you don’t have, follow the link below:
http://www.paramiko.org/

4. Make sure you have PyCrypto cryptography library. If you don’t have, follow the link below:
https://pypi.python.org/pypi/pycrypto

5. Make sure you have ecdsa cryptographic signature library if you are using Paramiko 1.12 or above. If you don’t have, follow the link below:
https://pypi.python.org/pypi/ecdsa/

6. Install fabric by typing the command below:

```
$ pip install fabric
```
